# Phosphorylation of TRIM28 Enhances the Expression of IFN-β and Proinflammatory Cytokines During HPAIV Infection of Human Lung Epithelial Cells

**DOI:** 10.3389/fimmu.2018.02229

**Published:** 2018-09-28

**Authors:** Tim Krischuns, Franziska Günl, Lea Henschel, Marco Binder, Joschka Willemsen, Sebastian Schloer, Ursula Rescher, Vanessa Gerlt, Gert Zimmer, Carolin Nordhoff, Stephan Ludwig, Linda Brunotte

**Affiliations:** ^1^Institute of Virology Muenster, Westfaelische Wilhelms-University Muenster, Muenster, Germany; ^2^Cluster of Excellence “Cells in Motion”, Westfaelische Wilhelms-University Muenster, Muenster, Germany; ^3^Research Group “Dynamics of Early Viral Infection and the Innate Antiviral Response”, Division Virus-Associated Carcinogenesis (F170), German Cancer Research Center (DKFZ), Heidelberg, Germany; ^4^Center for Molecular Biology of Inflammation, Institute of Medical Biochemistry, Westfaelische Wilhelms-University Muenster, Muenster, Germany; ^5^Institute of Virology and Immunology (IVI), Bern, Switzerland; ^6^Department of Infectious Diseases and Pathobiology (DIP), Vetsuisse Faculty, University of Bern, Bern, Switzerland

**Keywords:** influenza, TRIM28, KAP1, TIF1-beta, innate immunity, IFN-β, RIG-I, PKR

## Abstract

Human infection with highly pathogenic avian influenza viruses (HPAIV) is often associated with severe tissue damage due to hyperinduction of interferons and proinflammatory cytokines. The reasons for this excessive cytokine expression are still incompletely understood, which has hampered the development of efficient immunomodulatory treatment options. The host protein TRIM28 associates to the promoter regions of over 13,000 genes and is recognized as a genomic corepressor and negative immune regulator. TRIM28 corepressor activity is regulated by post-translational modifications, specifically phosphorylation of S473, which modulates binding of TRIM28 to the heterochromatin-binding protein HP1. Here, we identified TRIM28 as a key immune regulator leading to increased IFN-β and proinflammatory cytokine levels during infection with HPAIV. Using influenza A virus strains of the subtype H1N1 as well as HPAIV of subtypes H7N7, H7N9, and H5N1, we could demonstrate that strain-specific phosphorylation of TRIM28 S473 is induced by a signaling cascade constituted of PKR, p38 MAPK, and MSK1 in response to RIG-I independent sensing of viral RNA. Furthermore, using chemical inhibitors as well as knockout cell lines, our results suggest that phosphorylation of S473 facilitates a functional switch leading to increased levels of IFN-β, IL-6, and IL-8. In summary, we have identified TRIM28 as a critical factor controlling excessive expression of type I IFNs as well as proinflammatory cytokines during infection with H5N1, H7N7, and H7N9 HPAIV. In addition, our data indicate a novel mechanism of PKR-mediated IFN-β expression, which could lay the ground for novel treatment options aiming at rebalancing dysregulated immune responses during severe HPAIV infection.

## Introduction

Influenza A viruses (IAV) are the leading cause of annually recurring respiratory infections affecting millions of people worldwide. Infection by seasonal viruses is accompanied by mild to severe symptoms, such as fever, headache and dry cough but immunocompetent patients usually recover within 2–3 weeks. In contrast, infections with highly pathogenic avian influenza viruses (HPAIV), such as H5N1 often cause severe viral pneumonia as well as multiple organ failure and can lead to death, as exemplified by the “bird flu” outbreak in Hong Kong in 1997 with an overall mortality rate of 33% (1–3). Uncontrolled expression of type I and type II interferons (IFNs) and high levels of proinflammatory cytokines, such as TNF-α, IL-1ß, IL-6, und IL-8 due to a hyperinduction of the innate immune and inflammatory responses are the suspected reasons for HPAIV-induced immunopathology (reviewed in (4)). The underlying molecular mechanisms and signaling pathways, which are responsible for the increased and sustained expression of IFNs and proinflammatory cytokines during HPAIV infection are still not fully understood. However, a virus-induced imbalance of stimulatory and inhibitory factors, which normally regulate the controlled onset and resolution of immune responses, is hypothesized (5).

The innate immune response to IAV is rapidly initiated by pathogen recognition receptors (PRRs), such as RIG-I, which recognize viral RNA in the cytoplasm of infected cells and activate a signal transduction cascade involving the adaptor protein MAVS and the transcription factors IRF3/7. Upon phosphorylation, IRF3/7 dimerize and translocate into the cell nucleus where they bind to the IFN-α/β promotor and facilitate gene transcription. Alternatively, membrane associated toll-like receptors (TLRs) can detect viral glycoproteins or sense viral RNA in endosomal compartments and signal via the adaptor protein MyD88 resulting in the activation of IRF3/5/7 and subsequently in IFN-α/β expression (6, 7). Secreted IFN-α/β bind to the interferon-α/β receptor on neighboring cells resulting in STAT1/2 phosphorylation by the receptor-associated Jak/Tyk kinases (8). This mediates the nuclear translocation of STATs and upregulation of the expression of hundreds of interferon-stimulated genes (ISGs), among them antiviral proteins, chemokines and proinflammatory cytokines. This allows the recruitment and activation of immune cells at the site of infection. To resolve the ongoing immune reaction and prevent immunopathology, negative immune regulators, such as the recently identified death-associated protein kinase 1 (DAPK1) (9) and others interfere with further signal transduction and cytokine expression.

Here, we have identified the host factor and transcriptional corepressor Tripartite motif-containing 28 (TRIM28/KAP1/TIF1β) as a critical regulator of IFN-β, IFN-γ and cytokine expression during infection with HPAIV. TRIM28 belongs to the family of TRIM proteins (10) of which most members are involved in the regulation of the immune response to diverse viruses (11, 12). Like most of the TRIM family members, TRIM28 possesses E3 ubiquitin ligase activity located in its N-terminal RBCC-domain. Its C-terminus contains a rather unique arrangement of functional domains including a heterochromatin protein 1 binding domain (HP1 BD), a plant homeodomain (PHD) and a bromodomain (Bromo), which is only shared by the three other TRIM-family members TRIM24/TIF1α, TRIM33/TIF1γ and TRIM66/TIF1δ. All four proteins are known for their function as transcriptional regulators and constitute the TRIM subfamily VI (13–15).

Functionally, TRIM28 is described as a universal genome regulator involved in embryonic and stem cell development, cell cycle regulation, apoptosis, cancer, diverse stress responses and immunity (16–18). Mice lacking TRIM28 die at an early embryonic stage emphasizing its crucial role during embryonic development (19). In addition, TRIM28 facilitates silencing of endogenous retroviruses (20), restricts pro-viral gene activation and suppresses lytic gene expression of Kaposi's sarcoma-associated herpes virus, Murine leukemia virus and human T-cell lymphotropic virus-1 (21–23). It possesses E3 SUMO ligase activity and interacts with diverse transcription factors and other proteins to modulate their activity. These functions of TRIM28 are suspected to be regulated by post-translational modification (PTM) including SUMOylation, phosphorylation and others, which often occur at acceptor sites located in close proximity to the functional domains in the C-terminus (24–26). In contrast to the majority of TRIM proteins, which comprise immune enhancing activities, TRIM28 is associated with immunosuppression (27). The protein was reported to downregulate the activity of several immune-related transcription factors, such as IRF7, IRF5 and IRF1 as well as STAT3 by varying mechanisms (28–30). A role of TRIM28 during IAV replication has not been investigated until today. First evidence for a possible functional relevance derives from a global SUMO-screening demonstrating that TRIM28 is deSUMOylated during IAV infection (31). Nevertheless, this study did not address whether TRIM28 is involved in the immune response to IAV infection.

In the present study, we demonstrate that TRIM28 is phosphorylated at serine 473 (S473), a site known to regulate TRIM28 corepressor activity, during infection of human lung epithelial cells with HPAIV. Furthermore, we establish a link of S473 phosphorylation to elevated IFN-β expression and provide compelling evidence that TRIM28 is a key factor in the development of cytokine overexpression during HPAIV infection. These results could be the starting point for the development of new immunomodulatory strategies targeting TRIM28 post-translational modification to control the expression of type I IFNs as well as proinflammatory cytokines.

## Material and methods

### Cells and viruses

Human alveolar epithelial cells (A549), African green monkey kidney epithelial cell (Vero), HEK293T, HEK293T-Phoenix and Madin-Darby canine kidney type II cells (MDCK-II) were cultivated in Dulbecco's modified Eagle's Medium (DMEM) (Sigma, Germany) supplemented with 10% fetal bovine serum (Merck, Germany) and 1% Penicillin/Streptomycin (P/S) (Merck, Germany) at 37°C and 5% CO_2_. Human Umbilical Vein Endothelial Cells (HUVECs) were isolated from umbilical cords by dispase treatment and cultured on CellBIND® dishes (Corning, USA) in HUVEC-medium [50% EGM2 and 50% M199 (Biochrom, Germany) supplemented with 10% fetal calf serum (Sigma, Germany), 30 μg/mL gentamycin (Cytogen, Germany), 15 ng/mL amphotericin B (Biochrom, Germany), 100 IE Heparin (Ratiopharm, Germany), 2 mM L-glutamine (Lonza, Switzerland)] at 5% CO_2_ and 37°C. Upon infection HUVECs were cultured in M199 medium containing 1% BSA, 30 μg/ml gentamicin and 15 ng/ml amphotericin B. All work with HUVECs was conducted with the formal approval of the Ethics Committee of North Rhine-Westphalia and the University of Muenster. A/Thailand/KAN-1/2004 (H5N1) (KAN-1) was kindly provided by P. Puthavathana (Bangkok, Thailand). A/FPV/Bratislava/79 (H7N7) (FPV) was obtained from the virus depository of the Institute of Virology in Giessen, Germany. A/Hamburg/04/2009 (H1N1pdm) was a kind gift of the German National Reference Centre for Influenza (Brunhilde Schweiger, Berlin, Germany). A/Vietnam/1203/2004 (H5N1) (VN) and A/Anhui/1/2013 (H7N9) (Anhui) were kindly offered by Thorsten Wolff (RKI, Berlin). Recombinant A/Puerto Rico/8/34 (H1N1) (PR8), A/seal/Mass/1-SC35M/80 (H7N7) (SC35M) and A/WSN/33 (H1N1) (WSN) were generated using the pHW2000 reverse genetics system (32). All influenza viruses were propagated on MDCK-II cells in infection medium [DMEM supplemented with 1% P/S, 0.25% bovine serum albumin (BSA, Sigma) and 0.01% MgCl_2_ and CaCl_2_ (Roth, Germany)]. Infections were carried out by incubating cells in infection PBS (PBS supplemented with 1% P/S, 0.25% BSA and 0.01% MgCl_2_ and CaCl_2_) at the indicated multiplicity of infection (MOI) for 30 min. Experiments involving HPAIV were conducted in a biosafety level (BSL) 3 approved laboratory. Recombinant VSV (serotype Indiana) encoding firefly luciferase (VSV-luc) was generated by replacing the GFP gene in the previously described VSV-GFP vector by the firefly luciferase gene according to published procedures (33). VSV-luc was propagated on Vero cells and titrated by immunostaining with a rabbit polyclonal anti-VSV serum as described previously (34).

### Plasmids

Guide RNAs (gRNA) targeting TRIM28 and mCherry were designed with *Bbs*I overhang sequences. The gRNA oligonucleotides were ordered phosphorylated, annealed and ligated into *Bbs*I digested pSpCas9(BB)-2A-GFP plasmid (Addgene #48138) (35). For MyD88 and PKR, gRNAs were annealed, phosphorylated by PNK and cloned into *Bsm*BI digested lentiCRISPR v2 vector (Addgene #52961) (36). Oligonucleotide sequences are included in Supplementary Table S1. Full-length human TRIM28 was subcloned from pEGFP-TRIM28 (Addgene #45568) into *Not*I and *Xho*I (NEB, USA) digested pBluescript II SK(+/–) vector. In pBluescript II SK(+/–), TRIM28 mutants (S473A, S473E) were obtained by site-directed mutagenesis with non-overlapping primers. Subsequently, TRIM28 wildtype and the phospho-mutants were cloned into *Not*I and *Eco*RI (NEB, USA) digested retroviral vector pQCXIP. PCR primer sequences are included in Supplementary Table S2.

### Generation of knockout cells

A549 TRIM28 CRISPR-Cas9 knockout (KO) and control cells (Ctrl) were generated by transient transfection. In brief, A549 cells were transfected with either pSpCas9(BB)-2A-GFP harboring a gRNA targeting TRIM28 or a control gRNA targeting mCherry (plasmids were kindly provided by Nicole Fischer, Hamburg, Germany). Positively transfected cells were selected by fluorescence-activated cell sorting (FACS) and clonal cell lines were analyzed for TRIM28 KO by western blot. A549 PKR, RIG-I KO, MAVS KO and MyD88 KO cells were generated by lentiviral transduction as described elsewhere (9, 37). In brief, lentiviral particles were produced on HEK293T cells by transfection with the following three plasmids at a 3:1:3 ratio; (i) pCMV-DR8.91 (ii) pMD2.G (iii) lenti-CRISPR-vector. Virus particle-containing supernatants were harvested 48, 56, and 72 h post-transfection (h p.t.) and used for transduction of target cells. Successfully transduced cells were selected with 1 μg/ml puromycin (Sigma, Germany). Gene knockout in single cell clones was validated by western blot.

### Retroviral gene transfer

The empty retroviral vector pQCXIP or pQCXIP-TRIM28 expressing the different phospho-mutants were transfected into HEK293T-Phoenix packaging cells (Orbigen, USA). Retrovirus-containing supernatants were harvested 48 and 60 h p.t., supplemented with polybrene (Santa Cruz Biotechnology, USA) to a final concentration of 4 μg/ml and used for transduction of A549 TRIM28 KO cells. Transduced cells were selected with 1 μg/ml puromycin for 5 days to obtain stable cell lines and TRIM28 expression levels were analyzed by western blot. Stable TRIM28 mutant-expressing cells were subcloned to obtain single cell clones with equal expression of TRIM28 as measured by western blot.

### Cell treatments

Cells were treated with inhibitors for 1 h prior to infection, RNA transfection or induction of genotoxic stress. After removal of the inoculum, transfection mix or chemicals, inhibitors were added to the infection medium. The following inhibitors were used: ATM (KU-60019, Selleckchem, Germany), Chk2 (Chk2 inhibitor II, Abcam, Germany), p38 MAPK (SB202190, Calbiochem, USA), PKR (2-Aminopurine, Sigma, Germany), MEK (U0126, Taros Chemicals, Germany), MK2 (PF-3644022, Sigma, Germany), MSK1 (SB747651A, Axon Medchem, Netherlands) and the ROS-scavenging agent N-Acetyl-L-cysteine (NAC) (Sigma, Germany). Cells were stimulated by exposure to 1 kJ/m^2^ UVC-light using a Stratalinker 2400 UV Crosslinker (BioSurplus, USA), or incubation with H_2_O_2_, etoposide (Sigma, Germany) or IFN-β (R&D Systems, Germany) for the indicated times and concentrations. For RNA and HMW poly(I:C) (Invivogen, USA) stimulations, A549 cells were transfected using Lipofectamine 2000™ (Invitrogen, USA) according to the manufacturer's instructions. Therefore, total RNA from MDCK-II cells either infected with WSN at an MOI of 5 for 8 h or non-infected cells was isolated using the RNeasy Kit™ according to the manufacturer's instructions (Qiagen, Germany).

### MTT-assay

For cytotoxicity measurements, MTT [3-(4,5-dimethylthiazol-2-yl)-2,5-diphenyltetrazolium bromide] (Sigma, Germany) was added to the cells at a final concentration of 5 mg/ml for 4 h at 37°C and 5% CO_2_. As a positive control, 2 μM staurosporine (Sigma, Germany) was added for 10 h. Supernatants were aspirated and DMSO (Roth, Germany) was added for 5 min before the optical density (OD) was measured at a wavelength of 562 nm (MicroLumat Plus LB96V, Berthold Technologies, Germany).

### Western blot and antibodies

Cells were lysed with ice cold radioimmunoprecipitation assay (RIPA) buffer (25 mM TRIS pH 7.5, 150 mM NaCl, 0.1% SDS, 0.5% sodium deoxycholate, 1% Triton X-100) supplemented with the following protease and phosphatase inhibitors; 10 μM leupeptin (Sigma, Germany), 200 nM aprotinin (Roth, Germany), 5 mM benzamidine (Sigma, Germany), 2.5 mM pefabloc (Sigma, Germany), 10 mM beta-glycerophosphate (Sigma, Germany), 1 mM sodium orthovanadate (Sigma, Germany), 10 mM sodium fluoride (Roth, Germany) and 2.5 mM sodium pyrophosphate (Sigma, Germany). Lysates were sonicated for 20 s (pulse 50%, amplitude 30%) and pelleted at 4°C, 14.000 g for 15 min. Protein amounts were adjusted to 20 μg, mixed with 4 × sample buffer (0.25 M TRIS pH 6.8, 40% glycerol, 8% SDS, 10% β-mercaptoethanol, 0.01% bromophenol blue) and separated by SDS-PAGE. Proteins were transferred to nitrocellulose membranes and detected by using primary antibodies targeting tubulin (Sigma, Germany), PB1 (GeneTex, USA), TRIM28, TRIM28 S473-P, TRIM28 S824-P (Abcam, UK), CREB S133-P, RIG-I, eIF2α S51-P, ERK1/2, ERK1/2 T202/Y204-P, HSP27, HSP27 S82-P (Cell signaling Technologies, USA) and anti-mouse or anti-rabbit IgG secondary antibodies either conjugated to fluorophores (Licor, Germany) or horseradish peroxidase (Cell Signaling Technology, USA). Selected bands were densitometrically quantified using Licor Image studio software (Licor, Germany).

### Immunofluorescence

A549 cells were seeded on glass coverslips and fixed with 4% paraformaldehyde (Sigma, USA). Cells were permeabilized with 0.1% Triton X-100, and blocked for 30 min in 3% BSA. Slides were incubated overnight at 4°C with primary antibodies against IAV nucleoprotein (NP) (GeneTex, USA) and TRIM28 S473-P. Secondary antibodies anti-rabbit Alexa Fluor 488 (Invitrogen, USA) and anti-mouse Alexa Fluor 568 (Invitrogen, USA) were incubated for 1 h at room temperature. Cell nuclei were stained for 20 min with DAPI (Thermo Fisher Scientific, USA). Coverslips were mounted on glass slides in Mounting Medium S3023 (Dako Omnis, USA) and examined using a LSM-800 Airyscan confocal microscope (Carl Zeiss, Germany).

### Phosphoproteomic screen

A549 cells were stably labeled with “light” lysine (^12^C_6_, ^14^N_2_) and arginine (^12^C_6_, ^14^N_4_), “medium” lysine (^13^C_6_, ^14^N_2_) and arginine (^13^C_6_, ^14^N_2_) or “heavy” lysine (^13^C_6_, ^15^N_2_) and arginine (^13^C_6_, ^15^N_4_). Labeled cells were infected with either PR8, FPV or KAN-1 at an MOI of 5 for 2, 4, 6, and 8 h. “Light”-labeled cells were used as non-infected control (0 h), whereas “medium”- and “heavy”-labeled cells were infected for 2 or 6 h and 4 or 8 h, respectively. Lysates from non-infected, 2, 4 h infected cells (Mix 1) and non-infected, 6, 8 h infected cells (Mix 2) were subjected to tryptic digestion. Phosphopeptides were purified by cation exchange chromatography and TiO_2_-enrichment followed by LC-MS/MS analysis on a Proxeon Easy-nLC coupled to an LTQ-Orbitrap XL mass spectrometer. Data analysis was performed using Mascot and MaxQuant (v1.2.2.9) as previously described (38–40). Phosphorylation intensities of TRIM28 residues were quantified in relation to the phosphorylation of TRIM28 in non-infected cells in both lysate mixtures.

### Cytokine analysis

A549 TRIM28 KO and Ctrl cells were stimulated by transfection with 200 ng of viral or cellular RNA. The LEGENDplex™ Human Anti-Virus Response Panel (BioLegend Cat. No. 740350) was used for the simultaneous determination of the concentrations of IFN-α, -β, -γ, -λ1 and λ2/3 as well as IL-1β, IL-6, IL-8, IL-10, IL-12p70, TNF-α, IP-10, and GM-CSF in the supernatant. Cytokine capturing was performed according to the manufacturer's protocol in filter plates. Bead-bound cytokines were measured on a FACSCalibur Cytometer (Becton Dickinson) and concentrations were calculated using the LEGENDplex™ Data Analysis Software (BioLegend, USA).

### RNA isolation and quantitative real-time PCR (qRT-PCR)

RNA was isolated using peqGOLD TriFast™ according to the manufacturer's instructions (VWR, USA). Total RNA was reverse transcribed with oligo(dT) primers and RevertAid H Minus Reverse Transcriptase (Thermo Fisher Scientific, USA). RT-PCR was carried out in duplicates using a LightCycler® 480 II (Roche, Germany). Primer sequences are provided in Supplementary Table S3. Commercially available primers were used for analysis of IFN-β mRNA (Qiagen, Germany). Expression data were normalized to the housekeeping gene glyceraldehyde 3-phosphate dehydrogenase (GADPH) and analyzed using the 2^−ΔΔ*CT*^ method as described elsewhere (41).

### IFN-bioassay

A549 TRIM28 KO and Ctrl cells were stimulated by transfection of 250 ng of viral or cellular RNA and at 6 h p. t. supernatants were harvested. The cell-free supernatants were diluted 1:10 and added to Vero cells for another 16 h. Subsequently, Vero cells were infected with VSV-luc at an MOI of 5 for 5 h. Supernatants were aspirated, cells were lysed in passive lysis buffer (Promega, USA) and luciferase assay substrate (Promega, USA) was added. VSV-luc reporter gene expression was determined by measuring luminescence using a MicroLumat Plus LB96V luminometer (Berthold Technologies, Germany).

## Results

### Phosphorylation of TRIM28 is induced by HPAIV infection

**Figure 1:**
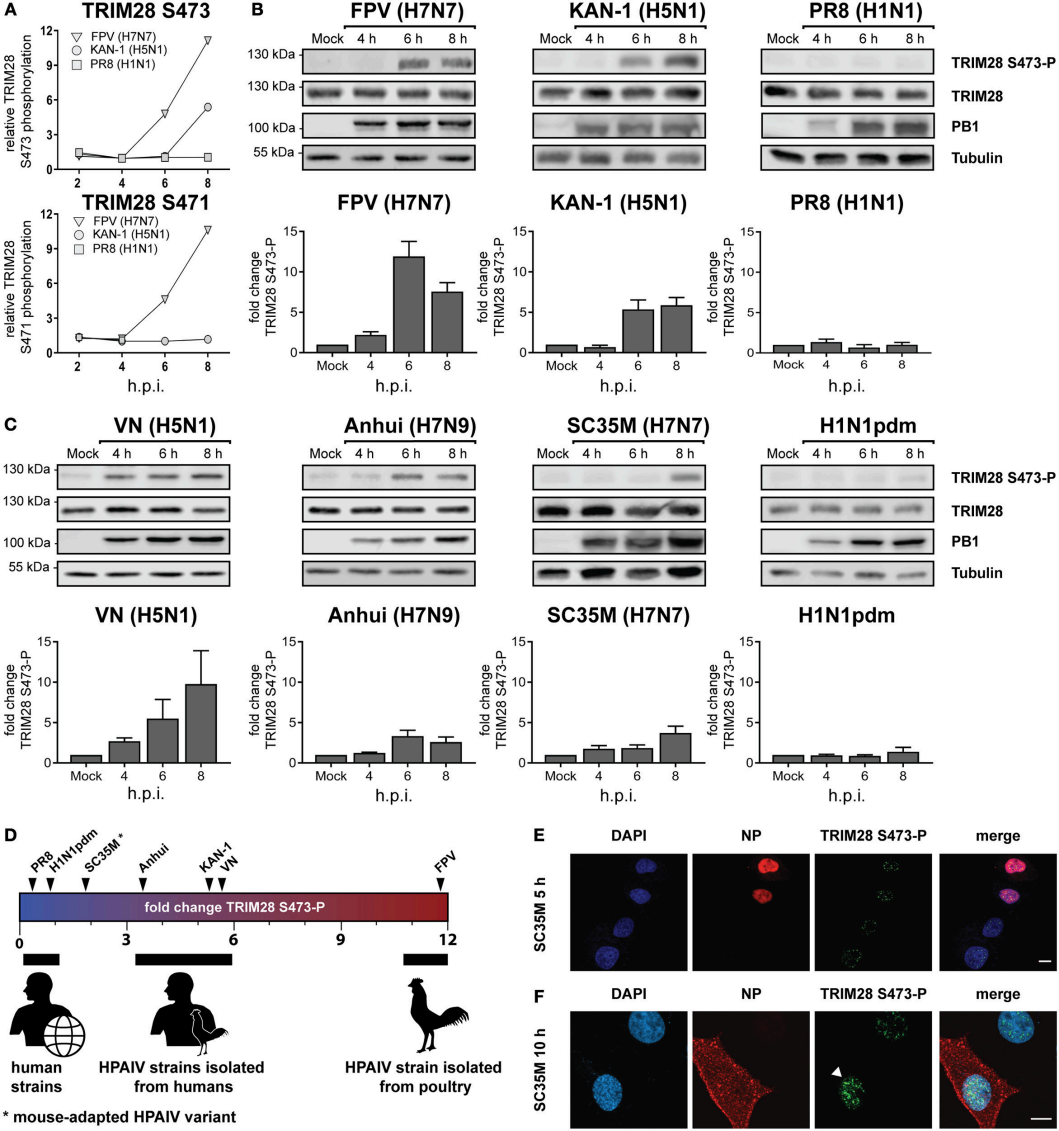
Phosphorylation of TRIM28 during HPAIV infection. **(A)** SILAC-labeled human A549 cells were infected with FPV (H7N7), KAN-1 (H5N1), and PR8 (H1N1) for the indicated times at an MOI of 5. Phosphorylated peptides were enriched and analyzed by mass spectrometry. The relative phosphorylation of TRIM28 at serine 473 (S473) and serine 471 (S471) are depicted. Western blot of A549 cells infected with **(B)** FPV, KAN-1, PR8 and **(C)** VN (H5N1), Anhui (H7N9), SC35M (H7N7), H1N1pdm at an MOI of 5 for the indicated time points. Phosphorylation of TRIM28 S473 was detected using a phospho-specific antibody. Detection of total TRIM28 and tubulin served as loading controls. Densitometric quantifications of S473 phosphorylation were normalized to tubulin intensities and are depicted as mean fold change (±SEM). **(D)** Schematic representation of mean fold changes of TRIM28 S473 phosphorylation compared to non-infected cells at 6 h p.i. A549 cells were infected with SC35M **(E)** at an MOI of 5 for 5 h or **(F)** at an MOI of 0.5 for 10 h. Nuclei were stained with DAPI. Viral nucleoprotein (NP) and TRIM28 S473 phosphorylation were stained using specific antibodies. Cells were analyzed by confocal laser scanning microscopy.

Viruses activate diverse signaling pathways in infected cells. To elucidate whether human adapted and highly pathogenic avian-derived IAV strains differentially activate kinase-governed signaling pathways a quantitative phosphoproteomic screen was performed (40). Human lung epithelial cells (A549) were infected with the human IAV strain A/Puerto Rico/8/34 (PR8, H1N1), the HPAIV strain A/Thailand/KAN-1/2004 (KAN-1, H5N1), which was isolated from a fatal human case following direct avian-to-human transmission and the HPAIV avian isolate A/FPV/Bratislava/79 (FPV, H7N7). This revealed that the host factor TRIM28 was increasingly phosphorylated at S473 during infection with KAN-1 and FPV but not with PR8 (Figure 1A, upper panel). For the neighboring serine 471 (S471), increased phosphorylation was only detected during FPV infection (Figure 1A, lower panel). These results were confirmed by western blot analysis using an antibody specific for phosphorylated TRIM28 S473 (Figure 1B). Based on these data, we speculated that TRIM28 phosphorylation could be a strain-dependent mechanism. To support this hypothesis, additional IAV strains were tested. We observed that TRIM28 S473 was also phosphorylated upon infection with the mouse-adapted HPAIV variant A/seal/Mass/1-SC35M/80 (SC35M, H7N7) and the HPAIV strains A/Vietnam/1203/2004 (VN, H5N1), A/Anhui/1/2013 (Anhui, H7N9) but not with the human-adapted 2009 pandemic H1N1 strain A/Hamburg/04/2009 (H1N1pdm) (Figure 1C upper panels). Quantitative western blot analysis further demonstrated that SC35M, KAN-1, and FPV induced S473 phosphorylation to different degrees, suggesting that all three strains have individual capacities to induce S473 phosphorylation (Figures 1B,C, lower panels). Plotting the virus strains according to the intensity of the induced S473-P signals indeed suggests that the degree of human adaptation inversely correlates with the capacity to induce S473 phosphorylation (Figure 1D). Like H5N1 viruses, H7N7 viruses can cross the species barrier from birds to humans and may cause severe to lethal respiratory disease in humans (42–44). As we observed S473 phosphorylation during infection with the mouse-adapted HPAIV variant SC35M, we used this strain as a representative for HPAIV in many experiments. This had the advantage that we could perform the experiments under BSL2 conditions. Interestingly, phosphorylation at S473 and S471 could be detected at 6 h p.i in the phosphoproteomic screen as well as in western blot analysis, indicating that it is not induced at an early stage of viral infection like viral entry or nuclear replication but rather at a later step. S473 phosphorylation was also observed at a low MOI of 0.1 (Supplementary Figure S1A). In addition, strain-dependent phosphorylation was also observed in primary HUVECs (Supplementary Figure S1B). Immunofluorescence data showed that the occurrence of nuclear S473 phosphorylation correlates with the cytoplasmic localization of the viral nucleoprotein (NP) 10 h after infection. In contrast, in cells infected for 5 h, only background phosphorylation was observed in the nucleus (Figures 1E,F). In summary, these results demonstrate that HPAIV of the H5N1, H7N7, and H7N9 subtypes induce phosphorylation of TRIM28 S473 at a late time point during infection. Furthermore, our data indicate that the capacity of IAV strains to phosphorylate TRIM28 inversely correlates with the degree of human adaptation.

### HPAIV-induced phosphorylation of TRIM28 is mediated by a signaling pathway not related to the DNA damage response (DDR)

**Figure 2:**
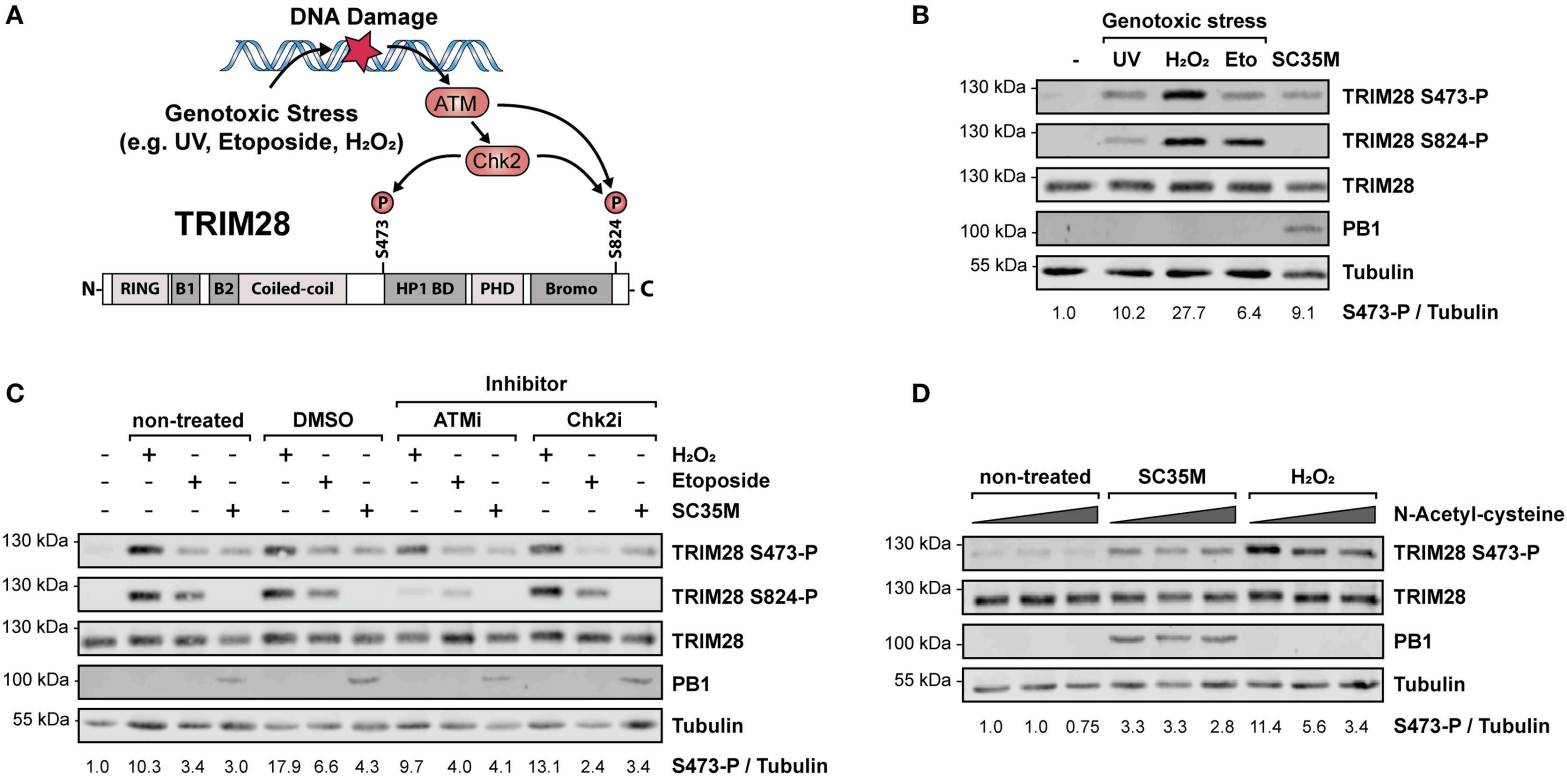
Phosphorylation of TRIM28 S473 occurs by non-DDR related kinases during HPAIV infection. **(A)** Schematic representation of the involved kinases leading to TRIM28 S473 and S824 phosphorylation during DNA damage response. **(B)** A549 cells were treated with 250 μM H_2_O_2_ for 3 h, 100 μM etoposide for 6 h, infected for 10 h with SC35M at an MOI of 20 or treated with 1 kJ/m^2^ ultraviolet light 30 min prior to western blot analysis. TRIM28 phosphorylation was detected using antibodies specific for TRIM28 phosphorylated at S473 or S824, respectively. Total TRIM28 and tubulin served as loading controls. Infection was validated using an antibody targeting the viral protein PB1. **(C)** A549 cells were treated with inhibitors for ATM (KU-60019, 2.5 μM), Chk2 (Chk2 Inhibitor II, 10 μM) and the solvent control (DMSO, 10 μM) 1 h prior to stimulation with H_2_O_2_, etoposide and SC35M infection in the presence of the corresponding inhibitors. **(D)** A549 cells were treated with increasing amounts of the reactive oxygen species scavenger NAC (5, 10, 15 mM) for 1 h following stimulation with 500 μM H_2_O_2_ for 2 h and infection with SC35M for 10 h at an MOI of 20 in the presence of NAC. TRIM28 S473 phosphorylation was monitored by western blot.

Phosphorylation of TRIM28 at positions S473 and serine 824 (S824) is widely described to occur in response to DNA damage and can be experimentally induced by various genotoxic stresses including treatment with H_2_O_2_, UV-radiation and etoposide (45–47). During DNA damage, phosphorylation at these sites is mediated by the kinase ataxia-telangiectasia mutated (ATM) and the checkpoint kinases 1 und 2 (Chk1/2) (48, 49) (Figure 2A). Phosphorylation is associated with different biological outcomes. While S473 is located in close proximity to the HP1 BD, which mediates the interaction with HP1 and repression of Krüppel-associated box zinc finger protein (KRAB-ZNF)-dependent genes, S824 lies next to the C-terminal bromodomain. Functionally, phosphorylation of S473 has been demonstrated to ablate binding of TRIM28 to HP1 and TRIM28-mediated repression of KRAB-ZNF-dependent genes. In contrast, S824 phosphorylation facilitates local chromatin relaxation (48) and, in combination with TRIM28 deSUMOylation, leads to the de-repression of DDR-responsive genes (25). Because infection with IAV has been reported to induce DDR (50, 51), we examined whether infection with SC35M induces the same phosphorylation pattern on TRIM28 compared to UV-radiation, H_2_O_2_ or etoposide treatment. Remarkably, we found that the induced phosphorylation patterns during IAV infection and DNA damage are different. Infection with SC35M induced phosphorylation of S473 but not S824 while all three genotoxic agents readily induced phosphorylation at both sites (Figure 2B). We further investigated whether ATM and Chk2 are also the responsible kinases for TRIM28 phosphorylation during IAV infection. Treatment of A549 cells with non-toxic concentrations of the inhibitors for ATM and Chk2 prior to stimulation with H_2_O_2_, etoposide or infection with SC35M clearly demonstrated that these kinases are not involved in IAV-mediated TRIM28 S473 phosphorylation (Figure 2C; Supplementary Figures S2A–D). Using NAC to scavenge reactive oxygen species (ROS) we could also exclude ROS as cause of TRIM28 S473 phosphorylation during SC35M infection (Figure 2D). In summary, these results demonstrate that during IAV infection TRIM28 S473 is not phosphorylated by the DDR-related kinases ATM and Chk2, which suggests that TRIM28 has a yet non-described, non-DDR related function in IAV infected cells.

### TRIM28 is a negative regulator of the innate immune response to IAV

**Figure 3:**
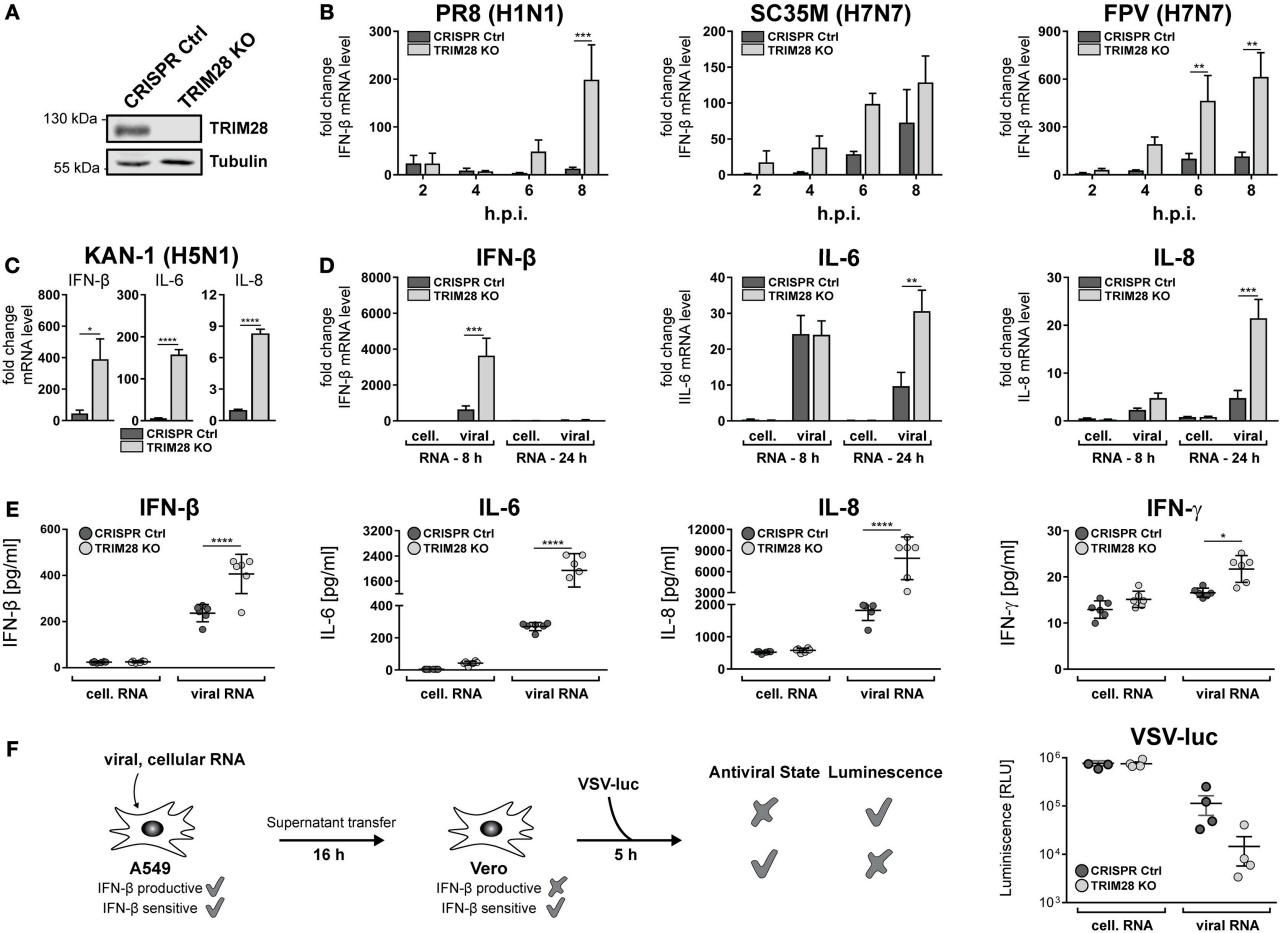
TRIM28 is a negative regulator of the innate immune response during influenza A virus infection. **(A)** Western blot of A549 TRIM28 KO cells. **(B)** Infection of TRIM28 KO cells with PR8, SC35M and FPV at an MOI of 5. Total RNA was isolated at the indicated time points and IFN-β mRNA levels were analyzed by qRT-PCR. IFN-β levels are depicted as mean *n*-fold change (±SEM) compared to non-infected cells. ^**^p ≤ 0.002; ^***^p ≤ 0.0002; two-way ANOVA; Sidak's multiple comparisons test. **(C)** TRIM28 KO cells were infected with KAN-1 (H5N1) at an MOI of 0.01 for 24 h. IFN-β, IL-6 and IL-8 mRNA levels were determined as described in **(B)**. ^*^p ≤ 0.03; ^****^p ≤ 0.0001; two-tailed unpaired *t*-test. **(D)** TRIM28 KO cells were transfected with 200 ng viral or cellular RNA. Total RNA was isolated 24 h p.t. and IFN-β, IL-6 and IL-8 mRNA levels were determined by qRT-PCR. Results are shown as mean *n*-fold change (±SEM) over non-treated cells. ^**^p ≤ 0.0021; ^***^p ≤ 0.0002; two-way ANOVA; Sidak's multiple comparisons test. **(E)** TRIM28 KO cells were treated as in **(D)**. Supernatants were analyzed using LEGENDplex™ bead immunoassay for the indicated cytokines at 24 h p.t. Results of six independent experiments are plotted as well as the mean (±SD). ^*^p ≤ 0.03; ^****^p ≤ 0.0001; two-way ANOVA; Tukey's multiple comparisons test. **(F)** A549 cells were transfected with 250 ng viral or cellular RNA for 6 h. Supernatants were harvested and transferred to Vero cells for 16 h. Stimulated Vero cells were infected with a luciferase-encoding vesicular stomatitis virus (VSV-luc) at an MOI of 5 for 5 h. Cells were harvested and virus replication was determined by luciferase assay. Results of four independent experiments are plotted as well as the mean (±SEM).

To gain insight into the general function of TRIM28 during viral infection, TRIM28 KO cells were generated using CRISPR-Cas9 (Figure 3A). Growth curve analyses demonstrated no pronounced effect on viral replication of SC35M and FPV in cells lacking TRIM28 compared to control cells (Supplementary Figures S3A,B). Because TRIM28 is described as a negative immune regulator, we analyzed the expression of IFN-β in these cells. Intriguingly, infection with PR8, SC35M or FPV resulted in elevated levels of IFN-β compared to infected control cells (Figure 3B). Elevated levels of IFN-β as well as the proinflammatory cytokines IL-6 and IL-8 were also observed during infection of TRIM28 KO cells with KAN-1 (Figure 3C). In addition, transfection of viral RNA (vRNA), as a trigger for the innate immune response, also resulted in higher mRNA levels of IFN-β, IL-6 and IL-8 in the absence of TRIM28 (Figure 3D). Importantly, we could also demonstrate that transcriptional upregulation correlated with significantly increased secretion of IFN-β, IL-6, IL-8 and IFN-γ in vRNA-treated TRIM28 KO cells at 8 and 24 h p.t. (Figure 3E; Supplementary Figures S4A,B). Because we did not observe an effect on SC35M and FPV replication, the biological function of increased IFN levels was manifested in an IFN-bioassay using a luciferase-expressing vesicular stomatitis virus (VSV-luc), which is highly sensitive to the action of IFNs. To induce an antiviral state, Vero cells were pre-treated with the supernatants from vRNA-stimulated TRIM28 KO and control cells. Infection with VSV-luc revealed a pronounced inhibition of viral replication in Vero cells that have been treated with the supernatant from stimulated TRIM28 KO cells compared to Vero cells treated with control cell supernatant, indicating that the increased IFN levels induced a more potent antiviral state (Figure 3F). In summary, these results demonstrate that TRIM28 functions as an important negative regulator of the expression of IFN-β, IFN-γ, IL-6 and IL-8 during IAV infection.

### Phosphorylation of TRIM28 S473 occurs in response to viral RNA but is independent of RIG-I

**Figure 4:**
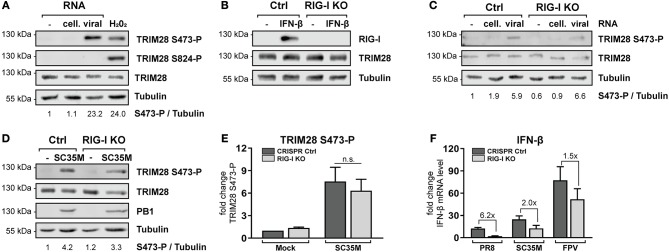
TRIM28 S473 phosphorylation is induced by viral RNA in a RIG-I independent manner. **(A)** A549 cells were transfected with 1 μg viral or cellular RNA. Lysates were harvested 4 h p.t. and TRIM28 S473 and S824 phosphorylation was analyzed by western blot. As a control, cells were treated with 250 μM H_2_O_2_ for 3 h. **(B)** Western blot of A549 RIG-I KO cells treated for 24 h with 500 U/ml IFN-β. **(C)** RIG-I KO cells were transfected with 500 ng viral or cellular RNA. TRIM28 S473 phosphorylation was monitored by western blot. **(D)** Western blot of TRIM28 S473 phosphorylation in RIG-I KO cells infected with SC35M for 10 h at an MOI of 20. **(E)** Densitometric quantifications of S473 phosphorylation in RIG-I KO cells infected as in **(D)**. TRIM28 S473-P levels were normalized to tubulin intensities. Results are plotted as mean *n*-fold change (±SEM) over mock-infected cells. n.s. *p* > 0.03; two-way ANOVA; Tukey's multiple comparisons test. **(F)** Infection of RIG-I KO cells with PR8, SC35M or FPV at an MOI of 5. Total RNA was isolated 8 h p.i. and IFN-β mRNA levels were measured by qRT-PCR. IFN-β levels are depicted as mean *n*-fold change (±SEM) over mock-infected cells.

The previous results demonstrated that TRIM28 negatively regulates the expression of IFN-β, IFN-γ, IL-6 and IL-8 during IAV infection. However, the role and biological function of S473 phosphorylation and the source of activation remained elusive. During IAV infection IFN-β is majorly expressed in response to sensing of viral RNA by cytosolic RIG-I (52, 53). Because our results demonstrate that TRIM28 is also involved in the expression of IFN-β, we speculated that TRIM28 S473 phosphorylation could be induced by a similar mechanism. Therefore, we analyzed whether transfection of vRNA induces S473 phosphorylation. We observed that TRIM28 S473 was markedly phosphorylated following transfection of vRNA or poly(I:C) (Figure 4A; Supplementary Figure S5A). Importantly, using RIG-I knockout cells (RIG-I KO) (Figure 4B), we could demonstrate that S473 phosphorylation during vRNA transfection (Figure 4C) and SC35M infection (Figures 4D,E) is retained in the absence of RIG-I. This provides evidence that S473 phosphorylation occurs independent of the RIG-I signaling pathway. To support the idea that RIG-I independent mechanisms contribute to the expression of IFN-β during infection with HPAIV, we infected wildtype and RIG-I KO cells with PR8, SC35M as well as FPV and measured the induction of IFN-β. This revealed that IFN-β expression was rather low in PR8 infected wildtype cells and seems to primarily depend on RIG-I as 6.2-fold less IFN-β was induced in the absence of RIG-I. In contrast, IFN-β was upregulated by 25-fold and 75-fold in SC35M and FPV infected wildtype cells, respectively. However, lack of RIG-I reduced IFN-β induction only by 2-fold in SC35M infected cells and 1.5-fold in FPV infected cells (Figure 4F). This indicates that the expression of IFN-β during FPV infection is not exclusively dependent on RIG-I but involves other signaling pathways. These data suggest that alternative RNA sensing receptors are responsible for the induction of S473 phosphorylation and contribute to the high levels of IFN-β during infection with HPAIV.

### Detection of viral RNA by the cytoplasmic RNA sensor PKR induces TRIM28 S473 phosphorylation

**Figure 5:**
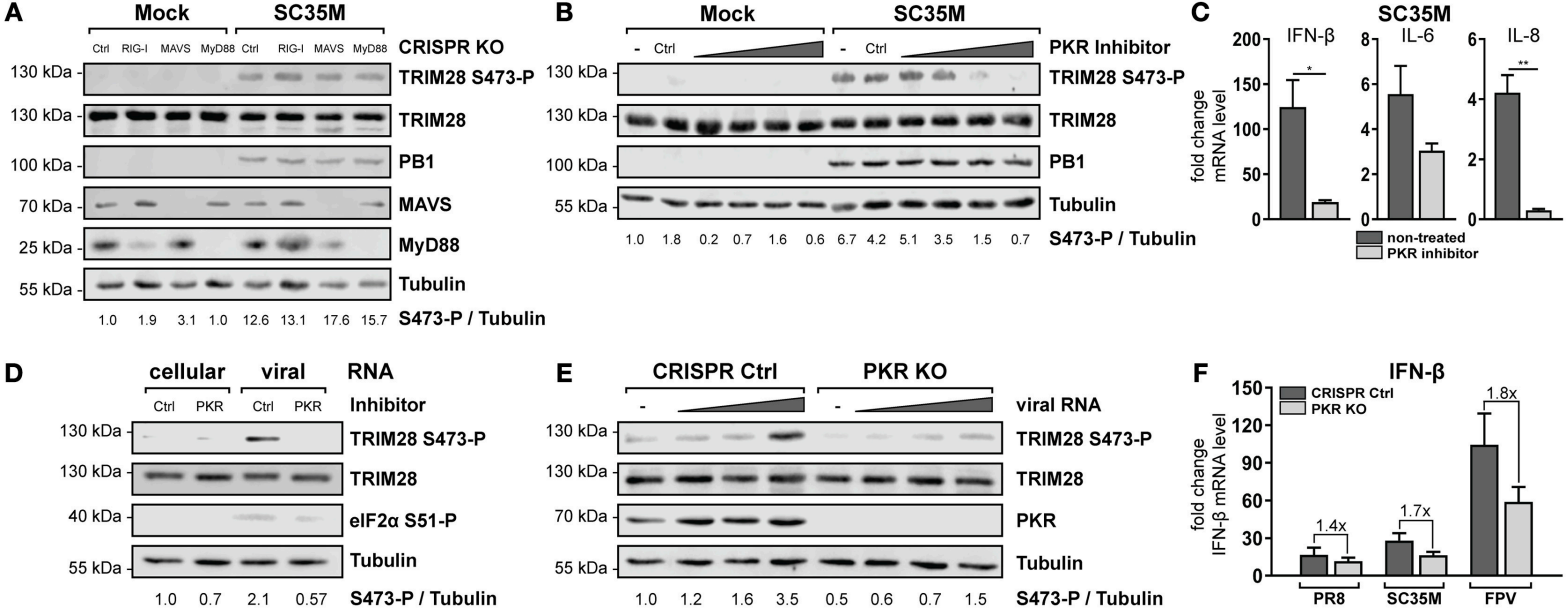
PKR inhibition ablates TRIM28 S473 phosphorylation. **(A)** A549 RIG-I, MAVS, and MyD88 KO cells were infected with SC35M at an MOI of 20 for 10 h. TRIM28 S473 phosphorylation was analyzed with a phospho-specific antibody by western blot. Total TRIM28 and tubulin served as loading controls. The viral protein PB1 was used to verify IAV infection. Expression of MAVS and MyD88 was detected using specific antibodies. **(B)** A549 cells were treated for 1 h with the PKR inhibitor 2-AP (0.5, 1, 5 and 10 mM). Subsequently, cells were infected with SC35M at an MOI of 20 for 10 h in the presence of 2-AP. **(C)** A549 cells were pre-treated with 10 mM 2-AP and subsequently infected with SC35M at an MOI of 0.1 for 24 h in the presence of 2-AP. mRNA levels of IFN-β, IL-6 and IL-8 were determined by qRT-PCR. Results are depicted as mean *n*-fold change (±SEM) over non-infected cells. ^*^p ≤ 0.03; ^**^p ≤ 0.002; two-tailed unpaired *t*-test. **(D)** A549 cells pre-treated with 10 mM 2-AP for 1 h were transfected with 500 ng viral or cellular RNA. Lysates were harvested 4 h p.t. and TRIM28 S473 phosphorylation was analyzed by western blot. PKR inhibition was controlled by detection of eIF2α S51 phosphorylation. **(E)** A549 PKR KO cells were transfected with 50, 250, and 500 ng viral RNA or 500 ng cellular RNA. Lysates were harvested 8 h p.t. and TRIM28 S473 phosphorylation was analyzed by western blot. **(F)** Infection of PKR KO cells with PR8, SC35M or FPV at an MOI of 5. Total RNA was isolated 8 h p.i. and IFN-β mRNA levels were measured by qRT-PCR. IFN-β levels are depicted as mean *n*-fold change (±SEM) over non-infected cells.

To further specify which immune recognition pathway comes into consideration for S473 phosphorylation and modulation of IFN-β expression during HPAIV infection, A549 cells lacking the adaptor proteins MAVS and MyD88 were examined. Infection with SC35M clearly demonstrated that TRIM28 S473 was still phosphorylated in cells lacking the RIG-I downstream effector MAVS, which supported the previous results obtained in RIG-I KO cells (Figure 5A, lane 7). Of note, RIG-I could not be detected in this western blot due to low induction by SC35M infection. However, RIG-I knockout in these cells was demonstrated following IFN-β treatment in Figure 4B. S473 phosphorylation was also retained despite lack of MyD88, which rules out the majority of TLRs as candidate receptors for mediating TRIM28 S473 phosphorylation (Figure 5A, lane 8). Another protein that is described to have RNA sensing capacity is the double-stranded RNA sensing protein kinase R (PKR), which also binds to double-stranded RNAs in the cytosol (54). Interestingly, inhibition of PKR using 2-Aminopurine (2-AP) impeded S473 phosphorylation in response to viral infection in a concentration dependent manner (Figure 5B) and following vRNA transfection (Figure 5D). Furthermore, Figure 5C shows that PKR inhibition also resulted in decreased levels of IFN-β, IL-6, and IL-8 during infection. As a genetic approach, A549 cells lacking PKR (PKR KO) were generated. Intriguingly, in these cells S473 phosphorylation after vRNA transfection was strongly reduced (Figure 5E). Infecting PKR KO cells with PR8, SC35M, and FPV revealed that the induction of IFN-β is differentially dependent on PKR. Although all three viruses induce less IFN-β in PKR KO cells, we observed a clear tendency that IFN-β induction is more dependent on PKR during infection with SC35M and FPV compared to PR8 (Figure 5F). This fits to our hypothesis that IFN-β induction in HPAIV but not PR8 infected cells is potentiated by a PKR activated signaling cascade. In summary, these results demonstrate that viral RNA sensing by PKR leads to TRIM28 S473 phosphorylation during HPAIV infection and presumably contributes to the high IFN-β levels.

### p38 MAPK and MSK1 phosphorylate TRIM28 s473 during HPAIV infection

**Figure 6:**
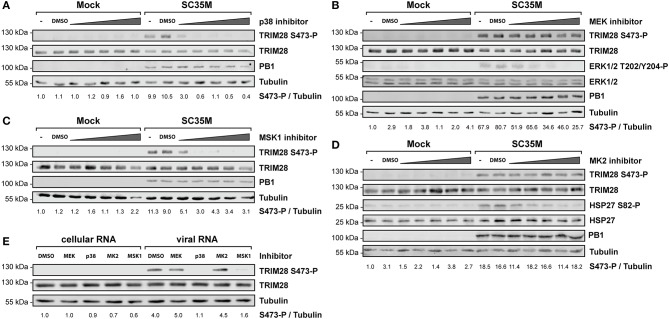
Inhibition of p38 and MSK1 ablates TRIM28 S473 phosphorylation. A549 cells were pre-treated with inhibitors for **(A)** p38 (SB202190), **(B)** MEK (U0126), **(C)** MSK1 (SB747651), and **(D)** MK2 (PF-3644022) at 0.1 μM, 0.5 μM, 1 μM, 5 μM and 10 μM before infection with SC35M at an MOI of 20 for 10 h and further incubation with the inhibitors. Lysates were examined with a phospho-specific antibody for TRIM28 S473 phosphorylation. Infection was verified by detection of the viral PB1 protein. Tubulin and total TRIM28 served as loading controls. MEK and MK2 inhibition was controlled by detection of ERK T202/Y204, and HSP27 S82 phosphorylation, respectively. **(E)** A549 cells were pre-treated with p38 (SB202190), MEK (U0126), MSK1 (SB747651) and MK2 (PF-3644022) inhibitors at 1 μM for 30 min. Pre-treated cells were transfected with 500 ng viral or cellular in the presence of the corresponding inhibitors. Lysates were harvested 4 h p.t. and TRIM28 S473 phosphorylation was analyzed by western blot.

**Figure 7:**
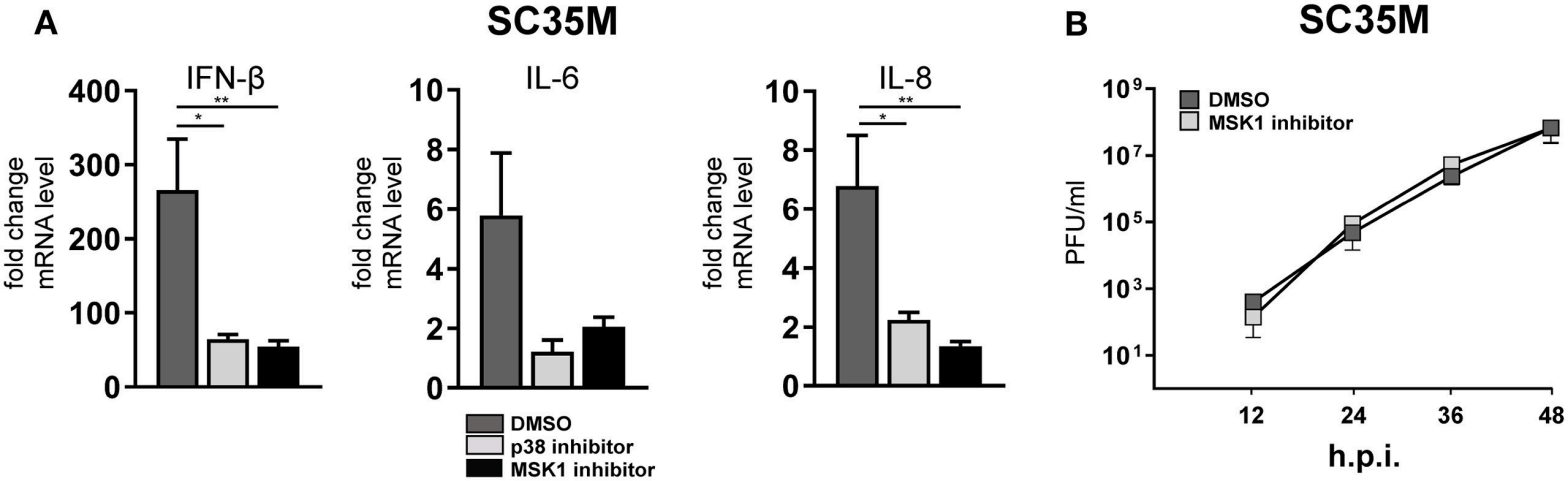
Inhibition of p38 and MSK1 downregulates IFN-β, IL-6 and IL-8 expression. **(A)** A549 cells were pre-treated with inhibitors for p38 (SB202190) and MSK1 (SB747651) and infected with SC35M at an MOI of 0.1 for 24 h in the presence of the inhibitors. IFN-β, IL-6 and IL-8 mRNA levels were determined by qRT-PCR. Results are depicted as mean *n*-fold change (±SEM) over non-infected cells. ^*^p ≤ 0.03; ^**^p ≤ 0.0021 one-way ANOVA; Dunnett's multiple comparisons test. **(B)** A549 cells were pre-treated with the MSK1 inhibitor SB747651 for 1 h and subsequently infected with SC35M at an MOI of 0.001. Supernatants were analyzed at the indicated time points by plaque assay.

In order to elucidate the signaling cascade responsible for TRIM28 S473 phosphorylation during viral infection, we concentrated further on kinases which are reported to be involved in the expression of IFN-β and proinflammatory cytokines during HPAIV infection and are known to be activated by PKR (55–57). This led us to investigate the stress inducible mitogen-activated protein kinase (MAPK) p38. Treatment of A549 cells with the p38 inhibitor SB202190 at specific and non-toxic concentrations efficiently blocked TRIM28 S473 phosphorylation during SC35M infection (Figure 6A; Supplementary Figure S6A) demonstrating that p38 plays a major role in this process. In contrast, treating cells with an inhibitor of MEK, thus blocking the ERK MAPK pathway, did not reduce S473 phosphorylation, excluding crosstalk from the classical MEK1/2-ERK1/2 MAP kinase pathway (Figure 6B; Supplementary Figure S6B). Well-described downstream kinases of p38 MAPK are MSK1 and MK2, which are both reported to be involved in the transcriptional regulation of cytokine expression (58, 59). Chemical inhibition of MSK1 but not MK2 resulted in the loss of S473 phosphorylation (Figures 6C,D; Supplementary Figures S6C,D). Importantly, inhibition of p38 MAPK and MSK1 led to reduced TRIM28 S473 phosphorylation during infection with the HPAIV KAN-1 and Anhui in primary HUVECs (Supplementary Figures S6F,G). This led us to conclude that MSK1 is the responsible kinase for S473 phosphorylation during IAV infection. Induction of S473 phosphorylation by transfection of vRNA was similarly abolished by inhibition of p38 and MSK1 but not by inhibiting MEK and MK2 (Figure 6E). Most importantly, loss of TRIM28 S473 phosphorylation by inhibition of p38 and MSK1 also resulted in decreased levels of IFN-β, IL-6, and IL-8 during infection with SC35M (Figure 7A), which was not caused by an inhibition of viral replication (Figure 7B). In conclusion, these data provide compelling evidence that TRIM28 S473 phosphorylation in response to PKR-dependent sensing of vRNA is mediated by the p38/MSK1-cascade during infection with HPAIV. Furthermore, these results strongly indicate that TRIM28 S473 phosphorylation results in enhanced expression of IFN-β and proinflammatory cytokines.

### Constitutive phosphorylation of TRIM28 S473 leads to increased induction of IFN-β, IL-6 and IL-8 during HPAIV infection

**Figure 8:**
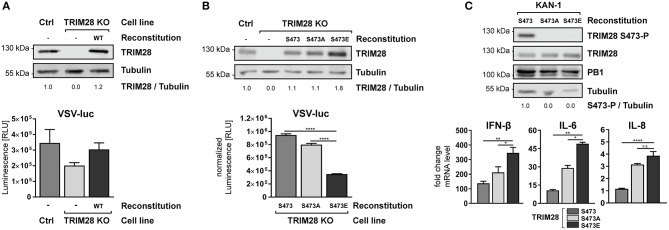
Constitutive TRIM28 S473 phosphorylation potentiates the innate immune response. **(A)** TRIM28 KO cells were stably reconstituted with wildtype TRIM28 by retroviral transduction. Reconstituted cells were infected with VSV-luc at an MOI of 0.01 for 15 h. Cells were harvested and luciferase activity was measured. **(B)** In addition to wildtype TRIM28, phospho-mimetic variants (S473A and S473E) were stably reconstituted in TRIM28 KO cells. VSV-luc infection was carried out as described in **(A)**. Results are depicted as mean RLU (±SEM) normalized to TRIM28 expression. ^****^p ≤ 0.0001; one-way ANOVA; Dunnett's multiple comparisons test. **(C)** Stably reconstituted TRIM28 KO cells were infected with KAN-1 at an MOI of 0.01. Total RNA was isolated 24 h p.i. and IFN-β, IL-6 and IL-8 mRNA levels were measured by qRT-PCR. mRNA levels are depicted as mean *n*-fold change (±SEM) over non-infected cells. n.s. *p* > 0.03, ^*^p ≤ 0.03; ^**^p ≤ 0.002; ^****^p ≤ 0.0001; one-way ANOVA; Dunnett's multiple comparisons test.

To establish the functional link between TRIM28 S473 phosphorylation and IFN-β expression, we reconstituted TRIM28 KO cells with either wildtype TRIM28 or the phospho-mutants S473A and S473E. Infection of TRIM28 KO cells with VSV-luc resulted in decreased viral replication. Most importantly, reconstitution of TRIM28 KO cells with the wildtype protein rescued VSV-luc replication (Figure 8A). Substitution of S473 with alanine (S473A) eliminates the phospho-acceptor site, while substitution with glutamic acid (S473E) mimics constitutive phosphorylation. As our previous data suggested that S473 phosphorylation regulates TRIM28-mediated repression of IFN-β, expression of these mutants should affect VSV-luc replication. Indeed, infection with VSV-luc demonstrated that reconstitution with TRIM28 S473E resulted in significantly decreased viral replication compared to cell expressing wildtype TRIM28 and TRIM28 S473A (Figure 8B). To proof that expression levels of IFN-β, IL-6, and IL-8 are also increased in the TRIM28 S473E expressing cells we infected the reconstituted cells with KAN-1 and performed qRT-PCR analysis. As seen in Figure 8C, the infected S473E expressing cells express higher levels of IFN-β, IL-6, and IL-8 compared to cells reconstituted with wildtype TRIM28 and TRIM28 S473A. Of note, also the non-phosphorylated form of TRIM28 harboring S473A showed increased levels of IFN-β, IL-6, and IL-8 compared to the cells expressing the wildtype protein. The reason for this is unknown. We speculate, that other phosphorylation sites, such as S471 and/or others, compensate for the lack of S473 phosphorylation. The phosphorylation dynamics of other phosphorylation sites of TRIM28 are not well-understood and require further investigation. In summary, our data demonstrate that S473 phosphorylation is functionally linked to increased expression of IFN-β, IL-6, and IL-8 and support our hypothesis, that phosphorylation at S473 modulates the corepressor activity of TRIM28 during infection with HPAIV.

## Discussion

Infection of humans with HPAIV is often associated with severe tissue damage and multiple organ failure caused by excessive production of IFNs and proinflammatory cytokines. The involved pathways as well as the underlying mechanisms leading to cytokine overexpression are not yet fully resolved. This knowledge gap impairs the development of new immunomodulatory treatment options due to the lack of suitable targets for efficient immunomodulatory therapies. Here, we report for the first time, that the cellular corepressor and negative immune regulator TRIM28 is the direct target of a signaling cascade involving the kinases PKR/p38/MSK1 during infection of human alveolar epithelial cells with HPAIV and contributes to the high expression levels of IFN-β, IL-6 and IL-8. Based on our results we hypothesize that TRIM28 is a key determinant for IFN-β overexpression and cytokine-mediated tissue damage and may represent a potential therapeutic target for the treatment of HPAIV-induced hypercytokinemia in humans.

TRIM28 is widely described as a genomic corepressor and negative immune regulator of cytokine expression in response to different immune stimuli. The described mechanisms of action involve its intrinsic E3 SUMO ligase activity as well as HP1-BD-mediated corepressor activity, which are assumed to be fine-tuned by SUMOylation and phosphorylation (24). The exact contribution of SUMOylation and phosphorylation to the regulation of TRIM28 activities has remained enigmatic. However, several reports have established an attractive regulatory model. While SUMOylation might control the general and genome wide repressor function of TRIM28, stimulus-dependent phosphorylation presumably regulates the de-repression of specific gene subsets (25) to allow stimulus- and stress-specific host responses. In line with this, Kubota et al. reported that tyrosine phosphorylation at positions Y449, Y458, and Y517 regulates HP1-binding and the controlled de-repression of genes required for stress tolerance and repair processes (60) and Li et al. demonstrated that phosphorylation of S824 regulates the expression of genes involved in cell cycle control and apoptosis in response to genotoxic stresses (25). Intriguingly, the authors of this report observed that the level of TRIM28 SUMOylation was decreased when S824 was mutated to aspartic acid to mimic constitutive phosphorylation, suggesting PTM crosstalk (25). Our own data suggest that phosphorylation of TRIM28 at S473 regulates the de-repression of IFN-β, IL-6 and IL-8. Nevertheless, we assume that additional sites could be involved, as we observed phosphorylation of the neighboring serine 471 in our phosphoproteomic screen (Figure 1A, lower panel). Supportive evidence for the biological relevance of phosphorylation of S473 and S471 comes from other proteomic studies in which these sites have been identified as phospho-acceptor sites (61, 62). In addition, both sites are also highly conserved in mice, rats and dogs, suggesting a biological important function. The p38/MSK1/TRIM28 signaling-axis was described previously to play a central role in myoblast differentiation. In these cells, TRIM28 phosphorylation controls the activity of the central transcriptional activator MyoD and thereby differentiation of myoblasts into myotubes (63). In addition, this report convincingly demonstrated that TRIM28 is a bona fide target of MSK1 in an *in vitro* kinase assay.

The detailed mechanism of TRIM28-mediated cytokine amplification during HPAIV infection remained unsolved. Based on available reports we assume that phosphorylation at S473 attenuates HP1- and chromatin-binding of TRIM28, which results in the loss of its corepressor function and leads to the de-repression of the described genes (49). However, other mechanism cannot be excluded. Because TRIM28 itself does not possess DNA binding activity, it is likely that cytokine repression occurs through the interaction with other transcription factors and chromatin remodeling enzymes. Indeed, TRIM28 was shown to interact and modulate the activity of diverse immune-related proteins, including NF-κB (64), STAT1 (28), STAT3 (29), IRF7 (30), and IRF5 (65). Nevertheless, a conjoint conclusion for the mode of action of TRIM28 is difficult to extract because diverse cell lines and immune stimuli were employed and the impact of S473 phosphorylation was not addressed. Thus, it needs to be investigated whether one of these factors facilitates TRIM28-mediated cytokine upregulation upon S473 phosphorylation. Recently, a novel model for TRIM28-mediated control of gene expression was proposed (66, 67). In this model, TRIM28 is involved in tethering of the 7SK snRNP complex to the promotor proximal regions of many rapid response genes that contain paused RNA Polymerase II (Pol II). Thereby, TRIM28 facilitates recruitment of the positive transcription elongation factor P-TEFb, which releases paused Pol II by phosphorylating serine 2 in the pol II C-terminal domain (CTD) and allows rapid elongation of transcription (62, 67, 68). Most intriguingly, TRIM28 was found to be associated with more than 13,000 promotor proximal regions, giving a rough estimation of how many genes might be regulated by TRIM28 (69). So far, the importance of S473 phosphorylation and SUMOylation for the control of immune-related genes has not yet been addressed in this model.

Phosphorylation of TRIM28 S473 was induced in a strain-dependent manner. This suggests that the degree of human adaptation as well as the reported characteristic to induce hypercytokinemia and tissue damage in humans might be determinants for TRIM28 phosphorylation during infection. To challenge this theory, we included the pandemic 2009 H1N1 virus in our analysis because it is a triple reassortant virus containing genes derived from humans, swine and birds and has acquired stepwise human adaptation in pigs prior to human transmission. In contrast to other pandemic IAV strains, H1N1pdm demonstrated weak virulence and low mortality rates (70) and human infections with this virus are not necessarily associated with hypercytokinemia and tissue damage. Thus, we expected that this strain would not trigger S473 phosphorylation. Indeed, we could not detect S473 phosphorylation with H1N1pdm, supporting our hypothesis (Figure 1C). The reasons for strain-dependent phosphorylation of TRIM28 on a molecular level are not known. It is tempting to speculate that it is mediated by virus intrinsic properties, such as avian specific protein signatures, differences in the NS1-mediated inhibition of PKR activation or other factors that underlie human adaptation. Alternatively, differences in replication speed or nuclear export of vRNPs, leading to accumulation of cytosolic vRNA cannot be excluded.

In addition to the novel role of TRIM28, our results suggest a new mechanism for PKR-mediated cytokine expression. Here, PKR senses viral RNA at a late time point during infection with HPAIV and provokes TRIM28 S473 phosphorylation via p38 and MSK1 with the consequence of excessive production of IFN-β, IL-6 and IL-8. PKR-mediated regulation of IFN-β expression in virus infected cells is described to be facilitated by activation of the translation elongation factor eIF2α as well as by compromised IFN-β mRNA stability (71, 72). Here we show, that in HPAIV-infected cells, PKR signals via p38/MSK1 to inactivate TRIM28 and potentiates the expression of IFN-β, IL-6 and IL-8 in human lung epithelial cells. At this moment, it remains unknown whether this pathway is also present in other IAV susceptible cell types, such as macrophages and dendritic cells, which could have severe immunopathological consequences as these cells are the main producers of IFNs and cytokines. The results from the phosphoproteomic screen as well as western blot analysis demonstrate that S473 phosphorylation occurs at a surprisingly late time point during infection. We assume that PKR activation requires the accumulation of viral RNA in the cytoplasm, possibly in the form of exported vRNPs, in order to boost IFN-β and cytokine expression through S473 phosphorylation. This mechanism of PKR activation has been previously suggested for Influenza B viruses (73) but is not described for IAV. Although our results convincingly show that TRIM28 phosphorylation is mediated by PKR, we can currently not exclude that other signaling pathways and receptors, such as TLR3, which signals independently of the adaptor proteins MyD88 and MAVS, are also involved.

**Figure 9:**
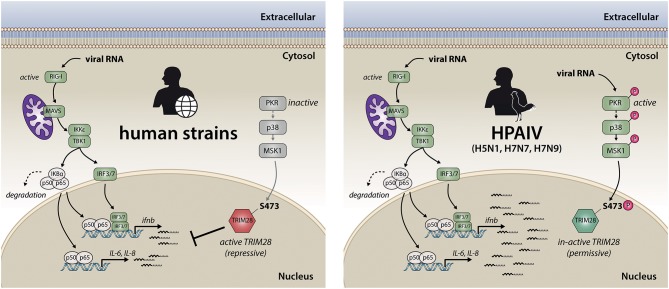
Model for TRIM28-mediated upregulation of IFN-β and proinflammatory cytokines during infection with the HPAIV subtypes H5N1, H7N9 and H7N7. Early during IAV infection, viral RNAs are sensed by RIG-I. This initiates a signaling cascade, which includes the adaptor protein MAVS and results in the dimerization and nuclear translocation of the transcription factors IRF3/7. During infection with human adapted strains, this leads to non-pathological levels of IFN-β and proinflammatory cytokines, which usually leads to the clearance of IAV infection (left). In contrast, during infection with the HPAIV of the subtypes H5N1, H7N7 and H7N9 (right), the expression of IFN-β, IL-6 and IL-8 is potentiated. This is facilitated by PKR-mediated sensing of viral RNA followed by signal transduction via p38 and MSK1 resulting in phosphorylation of the transcriptional corepressor TRIM28 at serine 473. This leads to the release of TRIM28 corepressor activity and finally results in elevated expression of IFN-β, IL-6 and IL-8, which is commonly associated with tissue damage und high mortality during HPAIV infections.

In summary, we propose a model for the TRIM28-mediated potentiation of cytokine expression during HPAIV infection. During infection with human adapted IAV strains, viral RNA is detected early during infection by RIG-I, which leads to the expression of non-pathological levels of IFN-β and proinflammatory cytokines (Figure 9, left side). In contrast, during infection with HPAIV of the H5N1, H7N7, and H7N9 subtypes cytosolic viral RNA is recognized by PKR, in addition to the RIG-I-dependent antiviral response. This leads to the activation of p38 and MSK1 and subsequently to phosphorylation of TRIM28 at S473 with the consequence of exacerbation of the ongoing immune response by amplification of IFN-β, IL-6 and IL-8 expression, which may lead to excessive immune cell recruitment and tissue inflammation (Figure 9, right side). We therefore propose, that controlling phosphorylation of TRIM28 by therapeutic interventions could prevent uncontrolled cytokine expression during HPAIV infections in humans.

## Author contributions

TK and LB are responsible for the concept and designed the experiments. TK, FG, VG, LH, SS, and CN conducted the experiments. MB and JW generated knockout cell lines. GZ generated recombinant VSV-luc. LB and TK wrote the manuscript. LB, TK, SL, and UR discussed and edited the manuscript.

### Conflict of interest statement

The authors declare that the research was conducted in the absence of any commercial or financial relationships that could be construed as a potential conflict of interest.
