# Supplementary material for: Phosphorylation of TRIM28 Enhances the Expression of IFN-β and Proinflammatory Cytokines During HPAIV Infection of Human Lung Epithelial Cells
Source: Front Immunol. 2018 Sep 28;9:2229. doi: 10.3389/fimmu.2018.02229 (PMC6172303; doi:10.3389/fimmu.2018.02229)

Supplementary Material

Phosphorylation of TRIM28 enhances the expression of IFN-β and proinflammatory cytokines during HPAIV infection of human lung epithelial cells.

Supplementary Table S1) Guide RNA sequences for CRISPR-Cas9 mediated gene knockout

gRNA DNA oligonucleotides	Sequence
TRIM28 fwd	5'- caccGACTACAGGCCGAGTGCAAAC -3'
TRIM28 rev	5'- aaacGTTTGCACTCGGCCTGTAGTC -3'
mCherry fwd	5'- caccGGGGTACATCCGCTCGGAGG -3'
mCherry rev	5'- aaacCCTCCGAGCGGATGTACCCC -3'
MyD88 fwd	5'- caccTGTCCCACGGACAGCAGAGC -3'
MyD88 rev	5'- aaacGCTCTGCTGTCCGTGGGACA -3'
PKR fwd	5'- caccGATGGAAGAGAATTTCCAGA -3'
PKR rev	5'- aaacTCTGGAAATTCTCTTCCATC -3'

Supplementary Table S2) Primer sequences used for cloning and site-directed mutagenesis

PCR Primer	Sequence
NotI-TRIM28 fwd	5'- gcttagcggccgcATGGCGGCCTCCGCGGCGGCA -3'
XhoI-TRIM28 rev	5'- taagcactcgagTCAGGGGCCATCACCAGGGCC -3'
EcoRI-TRIM28 rev	5'- taagcagaattcTCAGGGGCCATCACCAGGGCC -3'
SDM-TRIM28-S473A fwd	5'- cgccctcacctgcgcgggaccgttt -3'
SDM-TRIM28-S473A rev	5'- aaacggtcccgcgcaggtgagggcg -3'
SDM-TRIM28-S473E fwd	5'- gtcccgcgaaggtgagggcgagg -3'
SDM-TRIM28-S473E rev	5'- cctcaccttcgcgggaccgtttcacacc -3'

Supplementary Table S3 Primer sequences used for RT-qPCR

qPCR Primer	Sequence
huGAPDH fwd	5'- gcaaattccatggcaccgt -3'
huGAPDH rev	5'- gccccacttgatttggagg -3'
huIL6 fwd	5'- aacctgaaccttccaaagatgg -3'
huIL6 rev	5'- tctggcttgttcctcactagt -3'
huIL8 fwd	5'- cttgttccactgtgccttggtt -3'
huIL8 rev	5'- gcttccacatgtcctcacaacat -3'

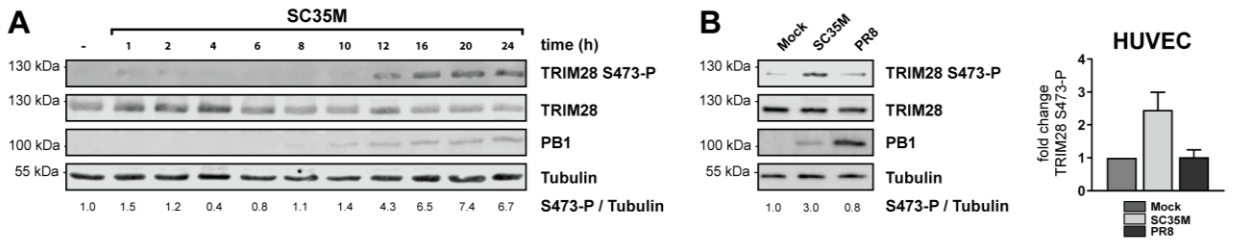

Supplementary Figure S1) (A) Western blot analysis of A549 cells infected with SC35M at an MOI of 0.1 for the indicated times. Phosphorylation of TRIM28 S473 was detected using a phospho-specific antibody. Detection of total TRIM28 and tubulin served as loading controls. The viral polymerase subunit PB1 was used to validate viral infection. **(B)** Primary HUVECs were infected with SC35M and PR8 at an MOI of 5 for 10 h. TRIM28 S473-P was analyzed by western blot. Densitometric quantifications of S473 phosphorylation of two different donors are shown. Values were normalized to tubulin intensities.

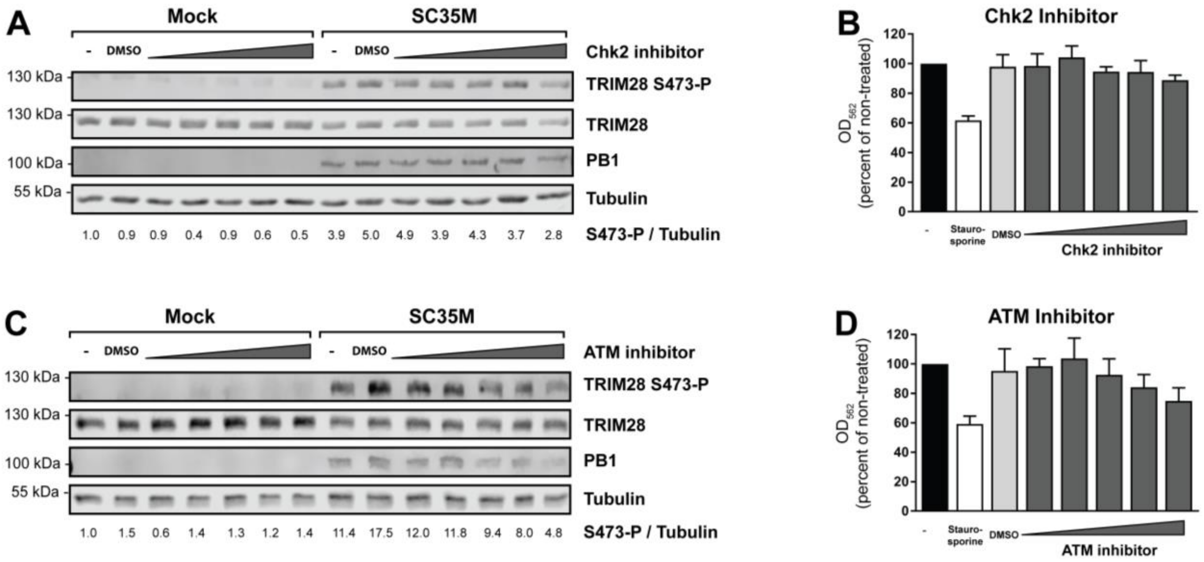

Supplementary Figure S2) (A) A549 cells were treated with inhibitors for **(A)** Chk2 (Chk2 inhibitor II), **(B)** ATM (KU-60019) at 0.1 µM, 0.5 µM, 1 µM, 5 µM and 10 µM before infection with SC35M at an MOI of 20 for 10 h in the presence of the inhibitors. Lysates were examined with a phospho-specific antibody for TRIM28 S473 phosphorylation. Infection was verified by detection of the viral PB1 protein. Tubulin and total TRIM28 served as loading controls. Cell toxicity of Chk2- **(C)** and ATM-inhibition **(D)** on A549 cells was determined. Cells were treated for 10 h with the indicated inhibitors and concentrations. Cell toxicity was measured with the MTT-assay. Results are depicted as mean OD_{562} (±SEM) compared to non-treated cells of three independent experiments.

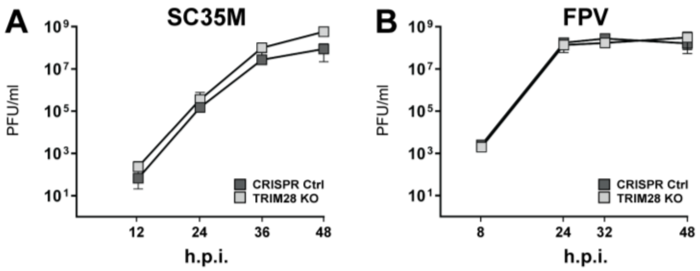

Supplementary Figure S3) A549 TRIM28 KO and Ctrl cells were infected with SC35M **(A)** and FPV **(B)** at an MOI of 0.001. Supernatants were harvested and analyzed for the release of infectious particles at the indicated times by plaque assay.

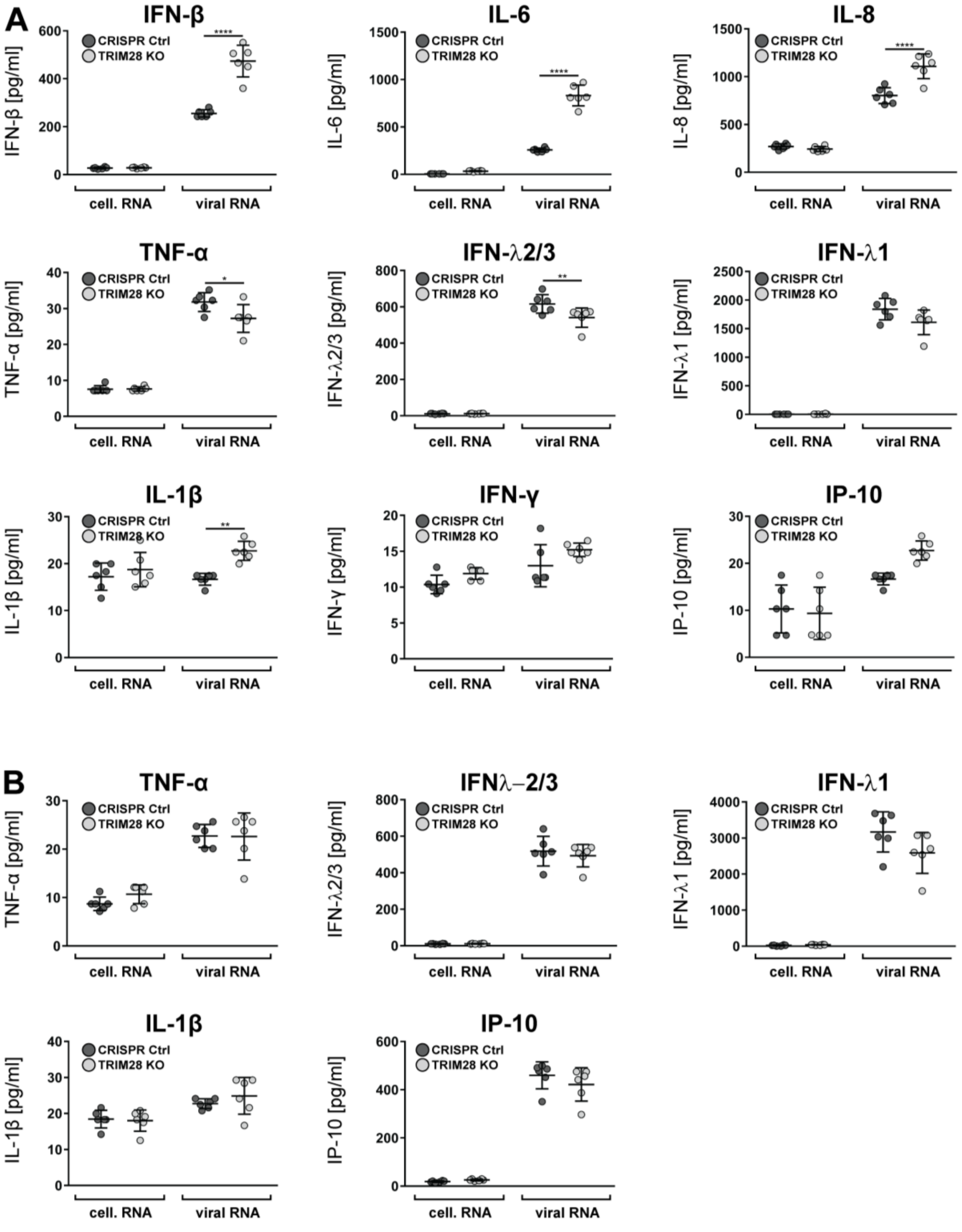

Supplementary Figure S4) A549 TRIM28 KO and Ctrl cells were transfected with 200 ng viral or cellular RNA. Cells were washed twice before medium was exchange 4 h p.t. Supernatants were analyzed using the LEGENDplex ™ bead immunoassay for the indicated cytokines at **(A)** 8 h p.t. and **(B)** 24 h p.t. Individual results of six independent experiments are plotted as well as the mean (±SD). *p≤0.03; **p≤0.002; ****p≤0.0001; two-way ANOVA; Tukey's multiple comparisons test.

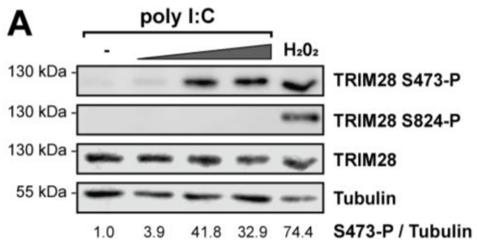

Supplementary Figure S5) (A) A549 cells were transfected with 50, 500 and 1000 ng poly(I:C) 4 h before harvesting and western blot analysis. Phosphorylation of TRIM28 was analyzed using phospho-specific antibodies to S473 and S824. TRIM28 and tubulin served as loading controls. As a control for TRIM28 phosphorylation, cells were treated with 250 µM H_20_2 for 3 h.

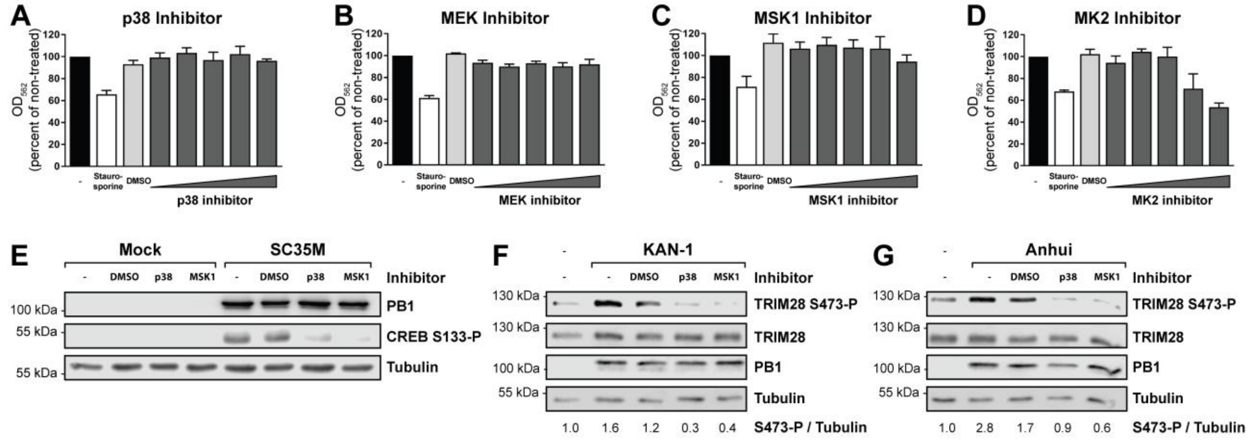

Supplementary Figure S6) A549 cells were treated with inhibitors for **(A)** p38 **(B)** MEK **(C)** MSK1 and **(D)** MK2 at concentrations of 0.1 µM, 0.5 µM, 1 µM, 5 µM and 10 µM for 10 h. Cell toxicity was determined by MTT-assay. Results are depicted as mean OD_{562} (±SEM) compared to non-treated cells of three independent experiments. **(E)** A549 cells were treated with inhibitors for p38 (SB202190) and MSK1 (SB747651) at 1 µM for 1 h. Subsequently, cells were infected with SC35M at an MOI of 20 for 10 h in the presence of the specific inhibitors before lysates were harvested and analyzed by western blot. Lysates were examined with a phospho-specific antibody for CREB S133 phosphorylation. Infection was verified by detection of the viral PB1 protein. Tubulin and total TRIM28 served as loading controls. Primary HUVECs were treated with inhibitors for p38 (SB202190, 10 µM) and MSK1 (SB747651, 5 µM) for 1 h. Subsequently, cells were infected with **(F)** KAN-1 and **(G)** Anhui at an MOI of 5 for 8 h in the presence of the inhibitors. TRIM28 S473 phosphorylation was analyzed by western blot.

Original western blots membranes

Figure 1 B + C

FPV Replicate I

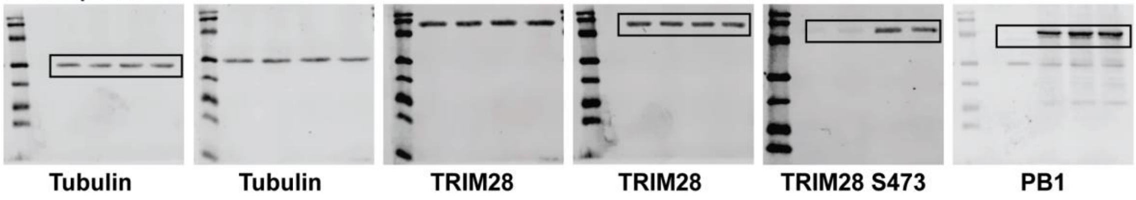

H1N1pdm Replicate I

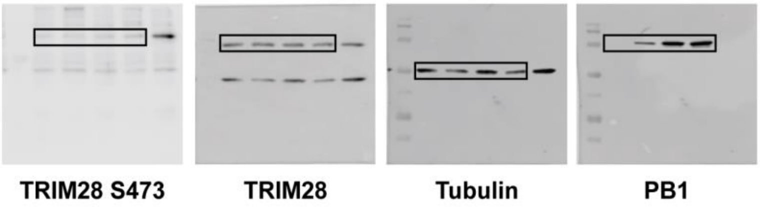

PR8/SC35M/KAN-1 Replicate I

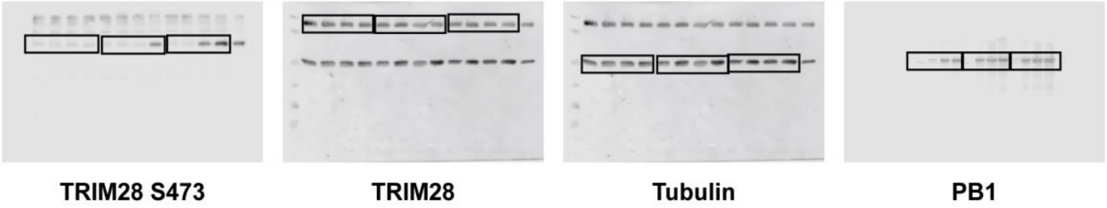

VN/Anhui Replicate I

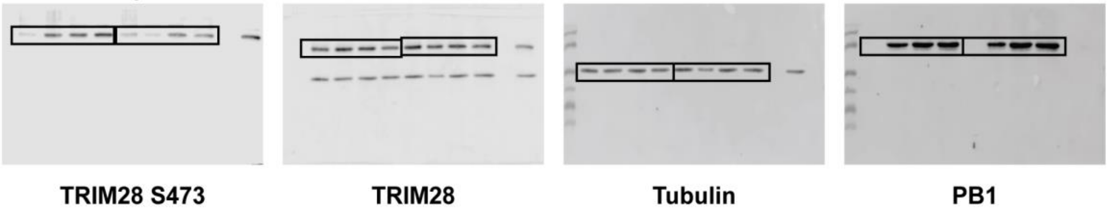

Figure 1 B+C Quantification Blots

PR8/SC35M/KAN-1/FPV 4 and 6 h Replicate I

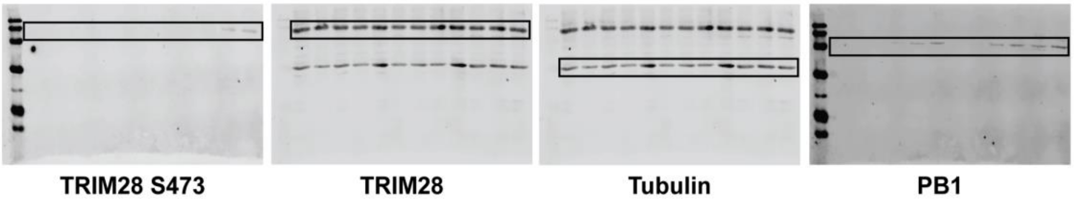

PR8/SC35M/KAN-1/FPV 8 h Replicate I

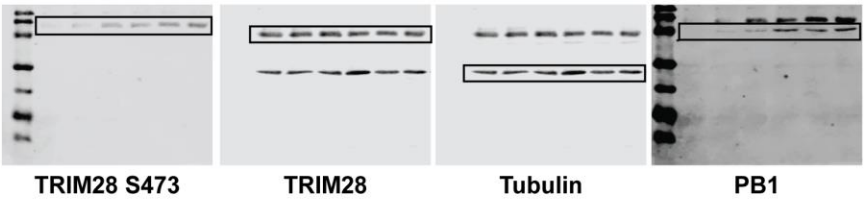

PR8/SC35M/KAN-1/FPV 8 h Replicate II

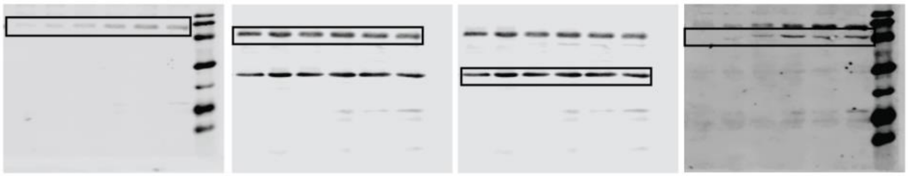

TRIM28 S473 | TRIM28 | Tubulin | PB1

PR8/SC35M/KAN-1/FPV 4 and 6 h Replicate III

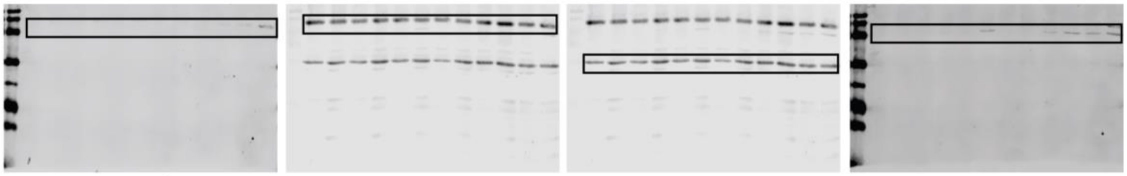

TRIM28 S473 | TRIM28 | Tubulin | PB1

PR8/SC35M/KAN-1/FPV 8 h Replicate III

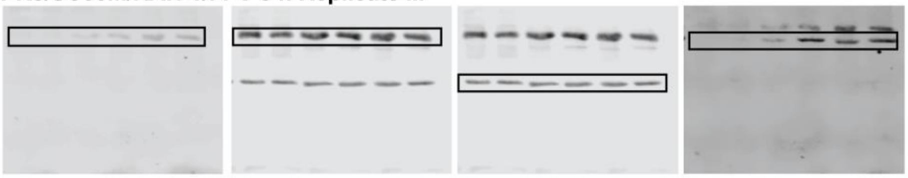

TRIM28 S473 | TRIM28 | Tubulin | PB1

H1N1pdm Replicate II + III

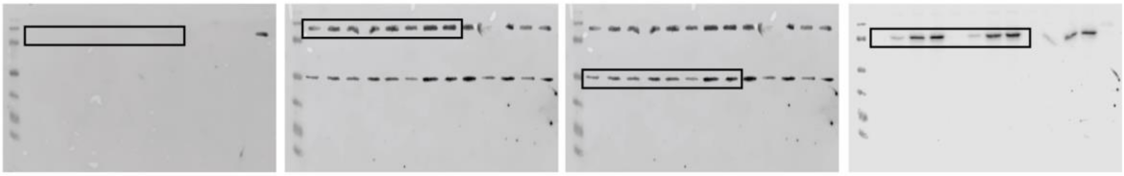

TRIM28 S473 | TRIM28 | Tubulin | PB1

PR8/SC35M/KAN-1/FPV 4 and 6 h Replicate II

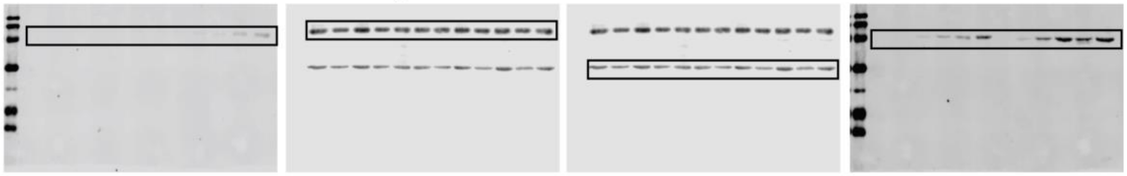

TRIM28 S473 | TRIM28 | Tubulin | PB1

VN/Anhui Replicate II

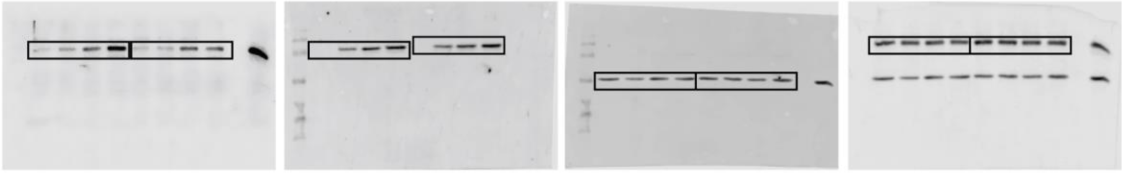

TRIM28 S473 | PB1 | Tubulin | TRIM28

VN/Anhui Replicate III

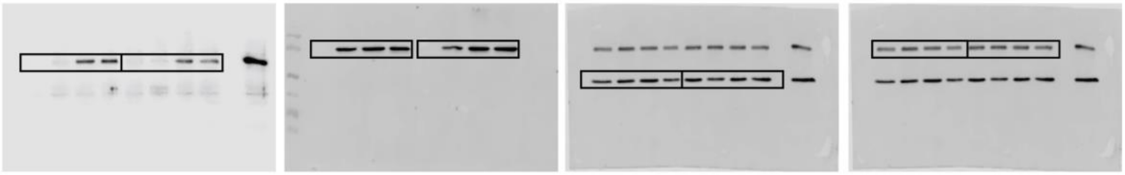

TRIM28 S473 | PB1 | Tubulin | TRIM28

Figure 2 B

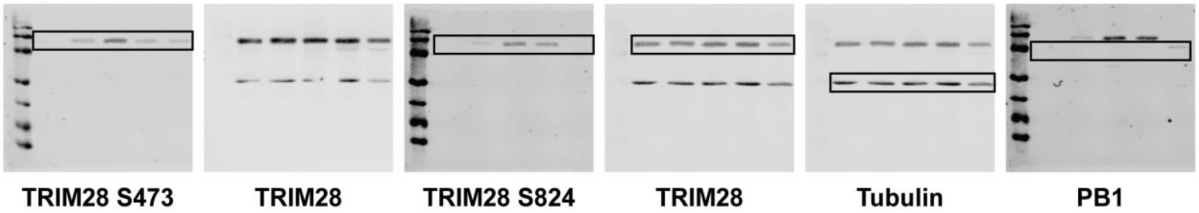

Figure 2 C

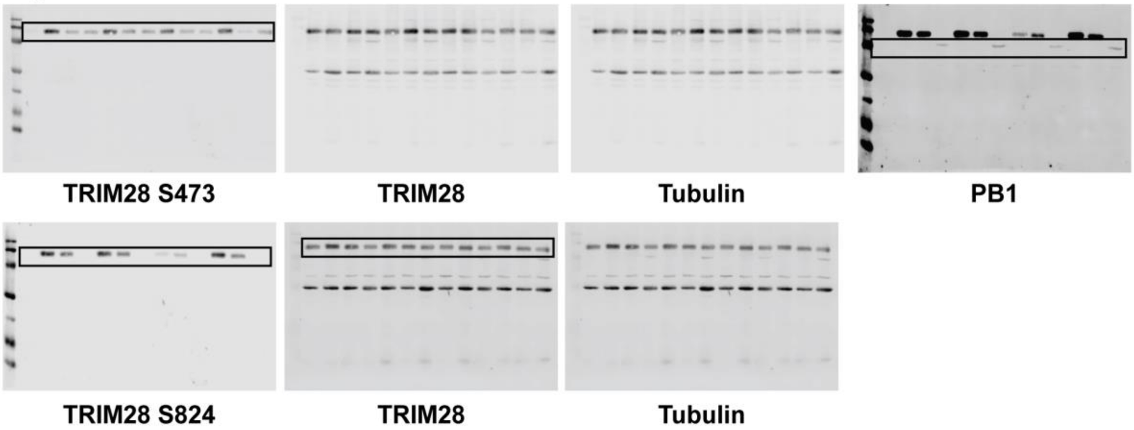

Figure 2 D

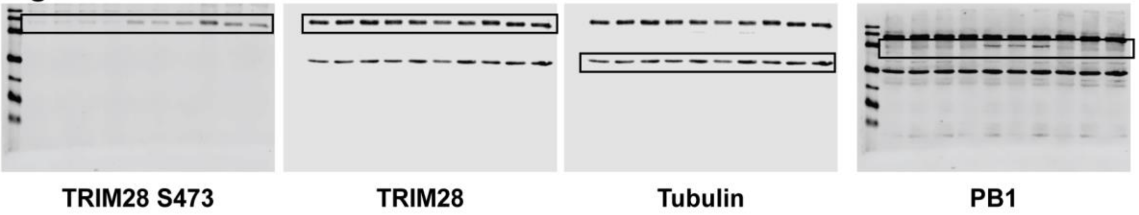

Figure 4 A

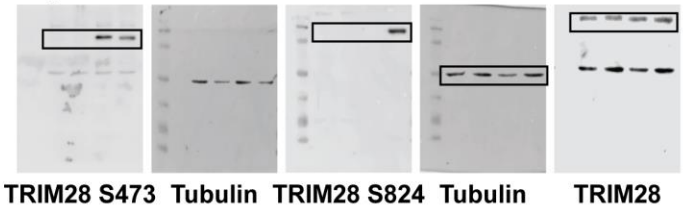

Figure 4 B

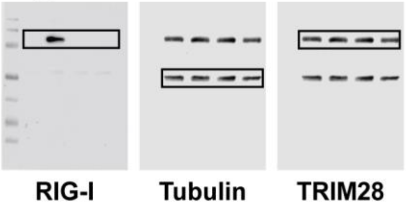

Figure 4 C

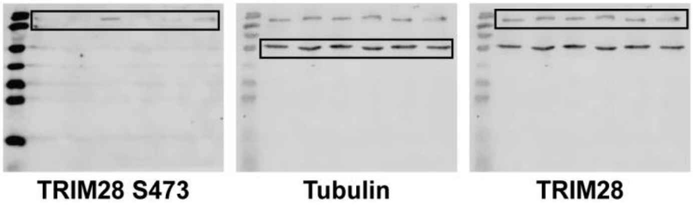

Figure 4 D

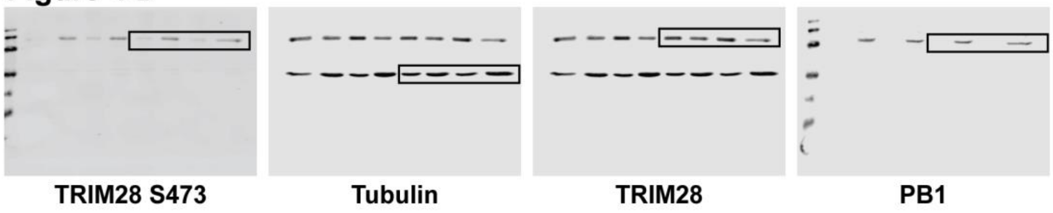

Figure 5 A

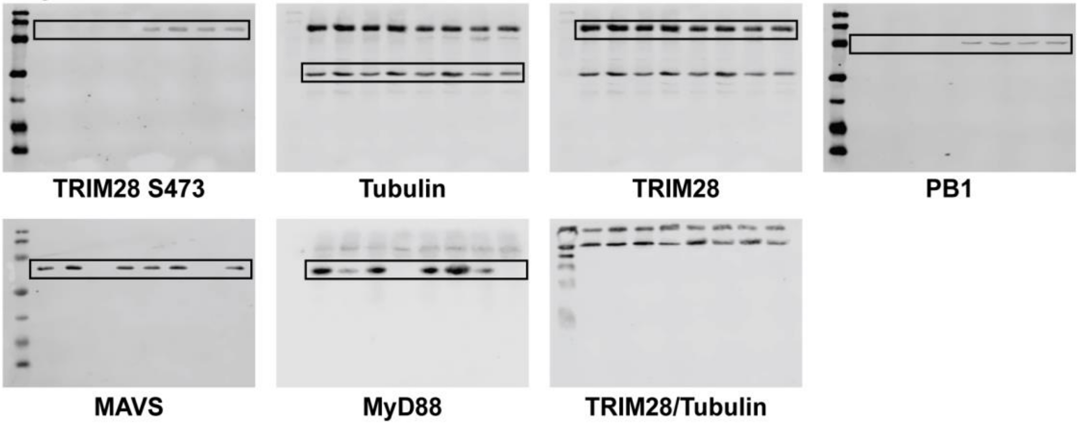

Figure 5 B

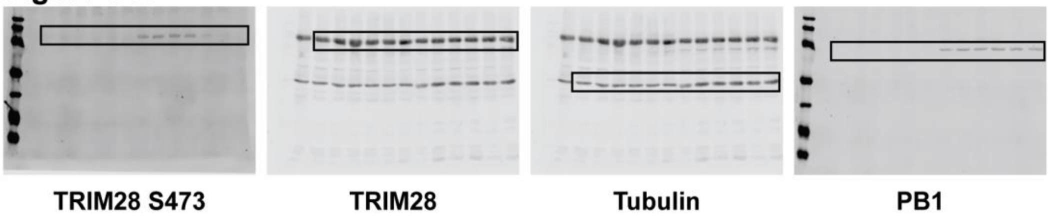

Figure 5 D

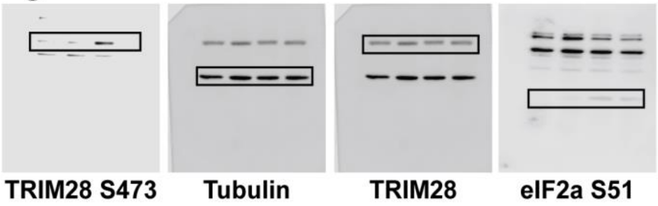

Figure 5 E

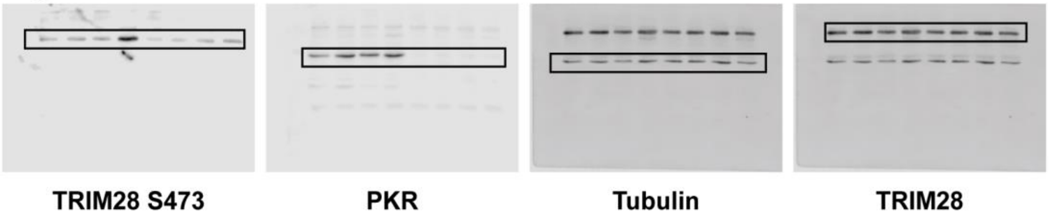

Figure 6 A

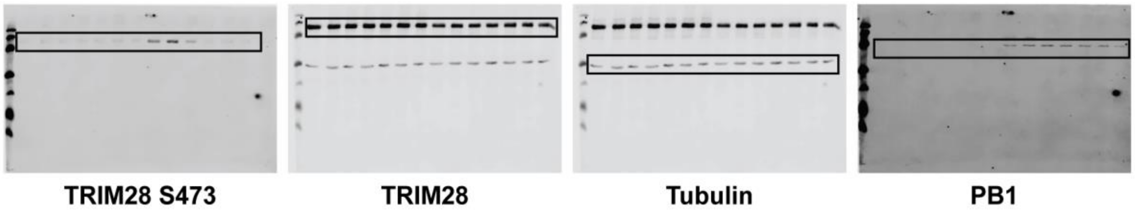

Figure 6 B

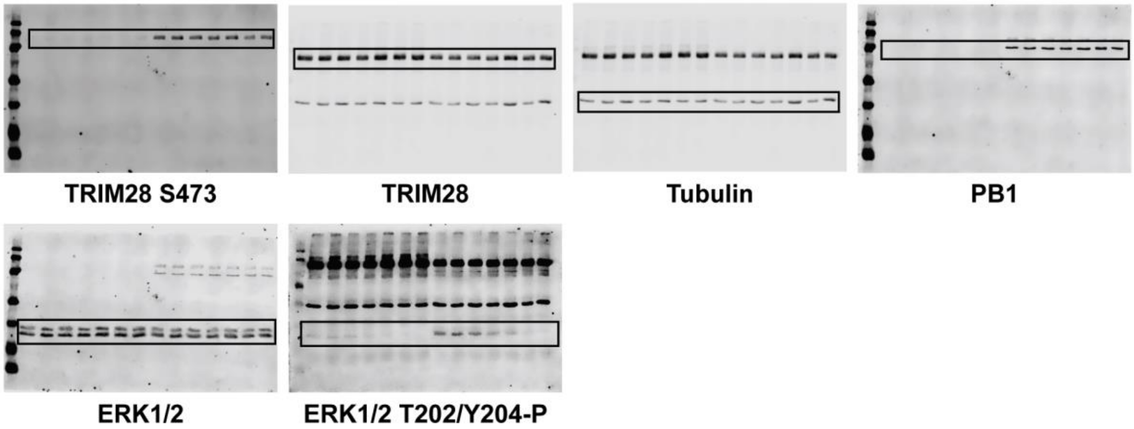

Figure 6 C

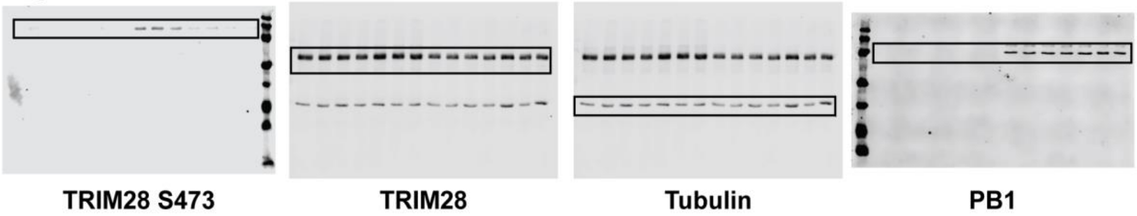

Figure 6 D

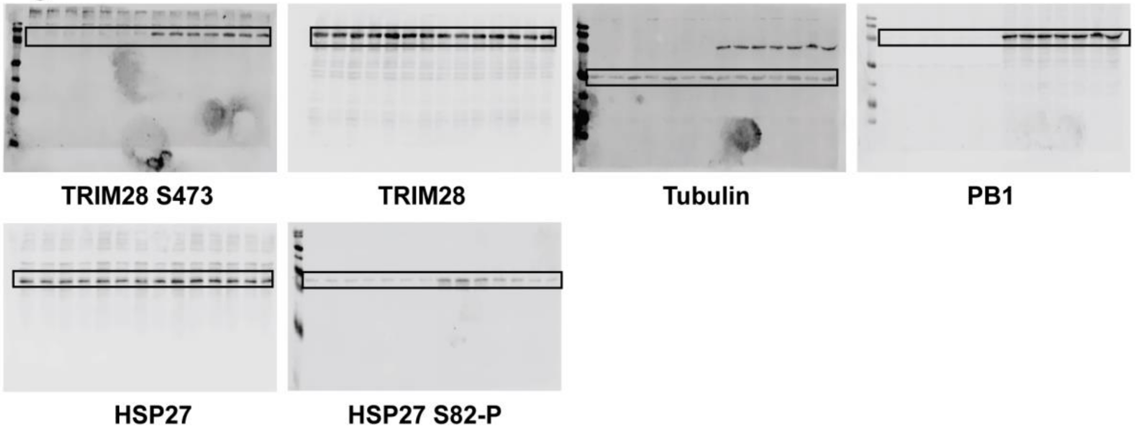

Figure 6 E

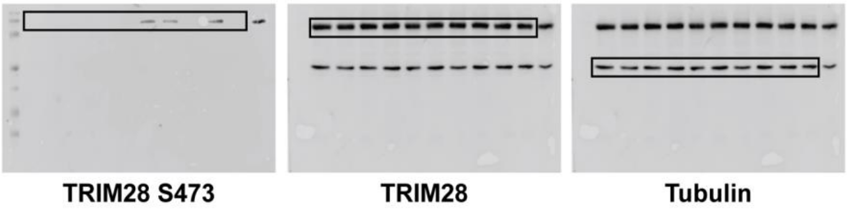

Figure 8 A

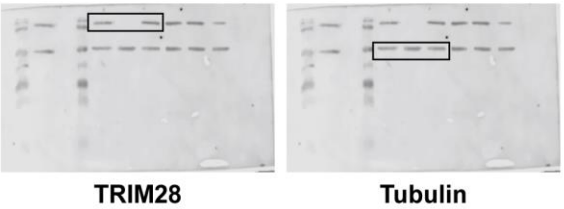

Figure 8 B

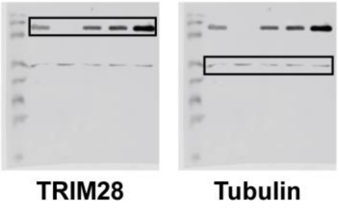

Figure 8 C

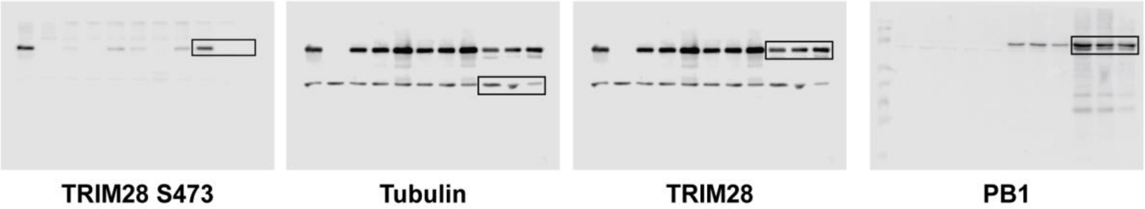

Figure S1A

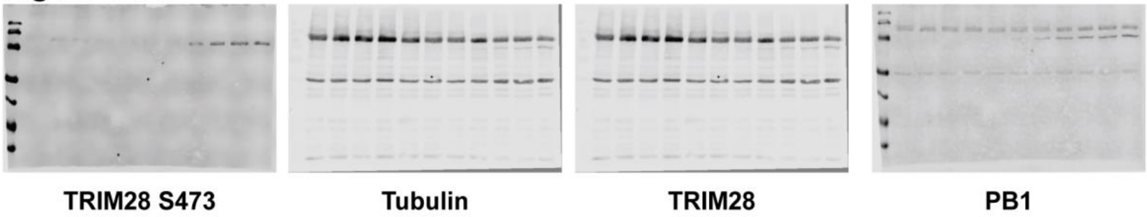

Figure S1B

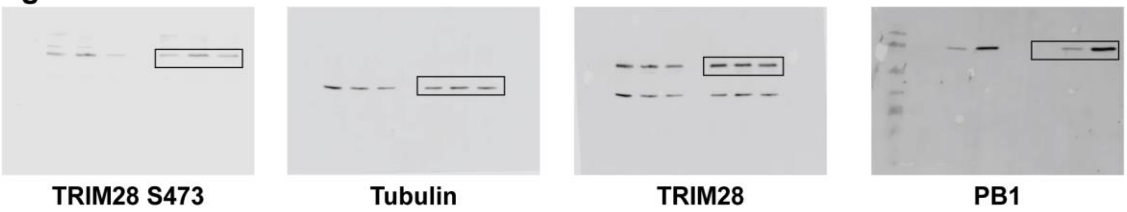

Figure S2A

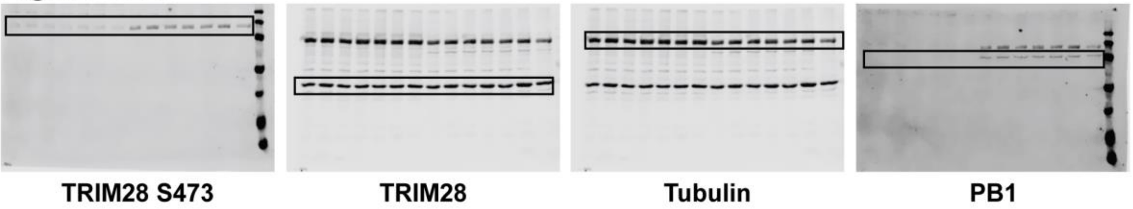

Figure S2C

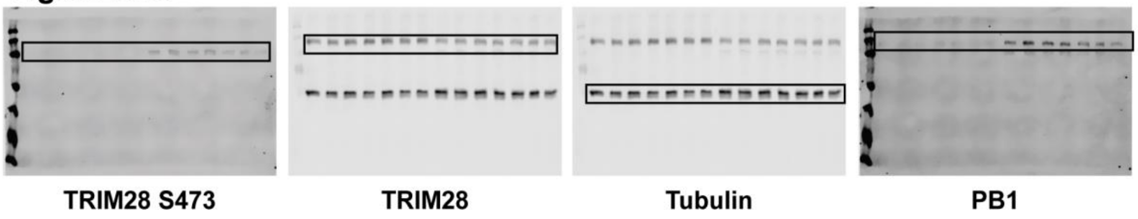

Figure S5A

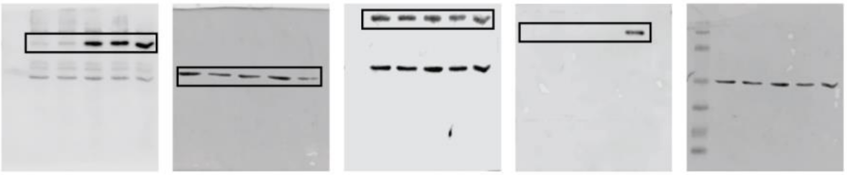

Figure S6E

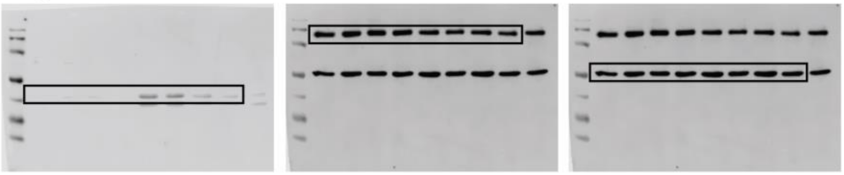

Figure S6F + G

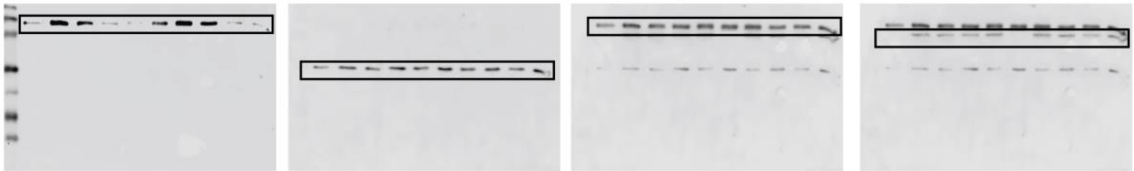

Supplement: Supplementary file 1 [file Image_1.pdf]
